# Extracellular adenosine modulates host-pathogen interactions through regulation of systemic metabolism during immune response in *Drosophila*

**DOI:** 10.1371/journal.ppat.1007022

**Published:** 2018-04-27

**Authors:** Adam Bajgar, Tomas Dolezal

**Affiliations:** Department of Molecular Biology and Genetics, Faculty of Science, University of South Bohemia in Ceske Budejovice, Ceske Budejovice, Czech Republic; Cornell University, UNITED STATES

## Abstract

Phagocytosis by hemocytes, *Drosophila* macrophages, is essential for resistance to *Streptococcus pneumoniae* in adult flies. Activated macrophages require an increased supply of energy and we show here that a systemic metabolic switch, involving the release of glucose from glycogen, is required for effective resistance to *S*. *pneumoniae*. This metabolic switch is mediated by extracellular adenosine, as evidenced by the fact that blocking adenosine signaling in the *adoR* mutant suppresses the systemic metabolic switch and decreases resistance to infection, while enhancing adenosine effects by lowering adenosine deaminase ADGF-A increases resistance to *S*. *pneumoniae*. Further, that ADGF-A is later expressed by immune cells during infection to regulate these effects of adenosine on the systemic metabolism and immune response. Such regulation proved to be important during chronic infection caused by *Listeria monocytogenes*. Lowering ADGF-A specifically in immune cells prolonged the systemic metabolic effects, leading to lower glycogen stores, and increased the intracellular load of *L*. *monocytogenes*, possibly by feeding the bacteria. An adenosine-mediated systemic metabolic switch is thus essential for effective resistance but must be regulated by ADGF-A expression from immune cells to prevent the loss of energy reserves and possibly to avoid the exploitation of energy by the pathogen.

## Author summary

The immune response is an energy-demanding process and a sufficient energy supply is important for resistance to pathogens. However, the systemic metabolism must be tightly regulated during an immune response since nutrients may also be exploited by the pathogen and host energy reserves are limited. Here we present how host-pathogen interaction can be influenced by extracellular adenosine. We show that adenosine regulates the allocation of energy during bacterial infections in flies and that its signal is crucial for host immunity. Furthermore, enhancing its effect may even boost host immunity during the acute phase. However, the removal of adenosine by adenosine deaminase and thus down-regulation of its effect on the energy metabolism might prevent unintended feeding of the pathogen at the expense of host energy reserves. Therefore, our work demonstrates on the one hand that immune cells usurp energy from the rest of the organism, which is crucial for the effectivity of the immune response but, on the other hand, that immune cells also regulate adenosine to prevent the negative consequences of the excessive release of energy.

## Introduction

Pro-inflammatory state of various immune cells in mammals, as neutrophils, dendritic cells and M1 macrophages and some adaptive-immunity cells (e.g. Th17), is associated with a metabolic switch including increased glycolysis and glucose consumption [1]. Therefore, the immune response is an energy-demanding process [2] and poor nutrition often leads to reduced immune resistance [3]. However, the energy supply to the immune system must be tightly regulated to protect it from exploitation by the pathogen [4] and also properly allocate limited energy reserves, especially because the immune response is known to be associated with anorexia [5,6]. For example, mycobacterial infection may lead to wasting due to the consumption of energy reserves [7].

The mechanisms for the systemic regulation of the metabolism during an immune response have only recently begun to be intensively studied. Systemic insulin resistance, caused by pro-inflammatory cytokines (*e*.*g*. TNF-α or Il-6), is believed to be an important mechanism to ensure the energy supply to the immune system [8]. Although this is likely to be beneficial during an acute response, chronic inflammation may lead to various metabolic disturbances [9]. Experimental studies of the effects of metabolic regulations on host-pathogen interactions are practically challenging, especially in higher experimental models such as mice [6,10]. Simpler model organisms such as the fruit fly *Drosophila melanogaster* may provide a more streamlined platform for such studies.

We have recently shown that extracellular adenosine (e-Ado) mediates a systemic metabolic switch upon infection of *Drosophila* larva by a parasitoid wasp [11]. This switch redirected energy normally devoted to developmental processes towards the immune system. Such a systemic switch is crucial for an effective immune response because blocking adenosine signaling drastically reduces resistance. We have also shown that immune cells produce this signal, thus usurping energy from the rest of the organism. Such a privileged behavior of the immune system was recently proposed to be important for an effective immune response [8].

Adenosine (Ado) is an important intracellular metabolite of purine metabolism. It can also appear extracellularly under certain conditions, thus becoming an important signaling molecule. For example, when tissue is damaged, adenosine triphosphate leaks out and is converted by ecto-enzymes into e-Ado [12]. Alternatively, when intracellular adenosine triphosphate levels decrease due to metabolic stress, increased adenosine monophosphate is converted to Ado, which is subsequently released into the extracellular space where it binds to adenosine receptors informing other tissues of this metabolic stress. During extensive exercise, muscles release Ado to induce fatigue to allow the initiation of recovery to this type of stress [13]. In another example, hypoxia induces adenosine to be released from the affected tissue to increase nutrient-rich blood flow [14]. This putative stress signaling by adenosine is conserved in evolution from primitive unicellular organisms all the way up to humans. For example, adenosine is released upon starvation by the social bacteria *Myxococcus xanthus* to induce the formation of fruiting bodies that produce spores for surviving harsh conditions [15]. Adenosine therefore represents a very ancient and universal system for signaling metabolic stress.

Here we investigate the regulatory role of e-Ado during the immune response and the effects of this regulation on host-pathogen interactions. Taking two complementary approaches, we block adenosine signaling by elimination of the adenosine receptor AdoR and enhance adenosine signaling by down-regulation of adenosine deaminase ADGF-A. We analyze the effects of these manipulations on host response to two types of bacterial infections: acute caused by *Streptococcus pneumoniae*, and chronic by *Listeria monocytogenes*. We show that adenosine regulates energy supply during bacterial infections in adult flies and that adenosine signaling is crucial for host defense, extending our previous results from research on parasitoid wasp infection [11]. In addition, we also demonstrate an important regulatory role of adenosine deaminase ADGF-A during the immune response. Enhancing adenosine effects may be beneficial for host resistance during the acute phase of the infection, but negative feedback regulation becomes important to prevent exhausting energy reserves and to prevent host nutrients being exploited by the pathogen.

## Results

### Phagocytosis is crucial for clearance of *S*. *pneumoniae*

**Fig 1:**
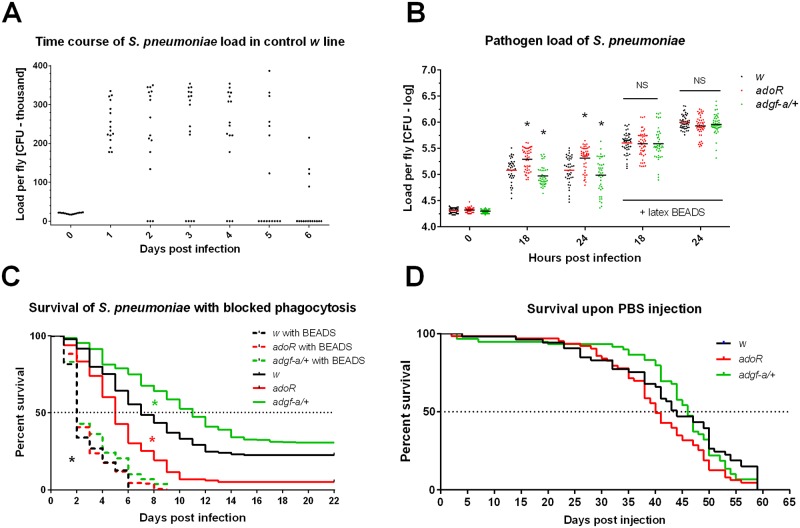
Host survival and pathogen loads during *Streptococcus pneumoniae* infection. (A) Pathogen loads of *S*. *pneumoniae* in colony forming units (CFU) per fly were determined over a span of 6 days in control *w* flies; each dot represents a load in one fly in linear scale (multiplied by thousand). Zero levels represent flies that cleared infection (majority at day 6). (B) Comparison of pathogen loads of *S*. *pneumoniae* at 18 and 24 hpi in flies with intact phagocytosis and in flies injected with latex beads (24 hours prior to infection) to block phagocytosis (labeled with “+ latex BEADS”). The *adoR* mutation (red) significantly increases and *adgf-a/+* (green) significantly decreases load compared to *w* control (black) in flies with intact phagocytosis both at 18 and 24 hpi (marked by asterisks). Blocking phagocytosis significantly increases pathogen loads for all genotypes compared to intact phagocytosis (significance not marked) with no significant differences between genotypes (marked as NS). Loads, shown in logarithmic scale (values follow lognormal distribution), were compared with unpaired t-tests. (C) Flies with phagocytosis blocked by latex beads displayed significantly reduced survival (dashed lines, marked by black asterisk) compared to flies with intact phagocytosis (solid lines) with equal reduction for all tested genotypes. With intact phagocytosis, *adoR* significantly decreases (marked by red asterisk) and *adgf-a/+* significantly increases (marked by green asterisk) survival compared to *w* control. (D) Survival of *w*, *adoR*, and *adgf-a/+* genotypes are comparable when injected with mock (PBS) buffer (*adoR* P = 0.1059 and *adgf-a/+* P = 0.5980 compared to *w*). Survivals were analyzed by both Log-rank and Gehan-Breslow-Wilcoxon tests.

When our control *w* flies were injected with 20 000 CFUs of *S*. *pneumoniae*, bacteria grew to 200–300 thousands within 24 hours (Fig 1A and 1B). This load persisted in the majority of flies for a few further days (acute phase) during which many flies died (Fig 1C). The surviving flies cleared the infection by the 5^th^-6^th^ day (Fig 1A), but some lethality still occurred after this, probably because they either did not clear the infection or they did not recover (Fig 1C). It has been previously demonstrated by injecting the flies with latex beads, which jams their hemocytes, making them unable to phagocytose, that phagocytosis was crucial for the clearance of *S*. *pneumoniae* [16]. We confirmed this result here when the injection of latex beads 24 hours before infection drastically increased pathogen load (to a million bacteria per fly; Fig 1B) and led to the rapid death of the flies within 2 days (Fig 1C). The effect of blocking phagocytosis on resistance was independent of the used genotypes (Fig 1B and 1C; see further detailed description of genotypes). Fig 1D shows the survival of PBS-injected (i.e. non-infected) flies under our experimental conditions.

### Systemic metabolic switch is required for an effective immune response during *S*. *pneumoniae* infection

**Fig 2:**
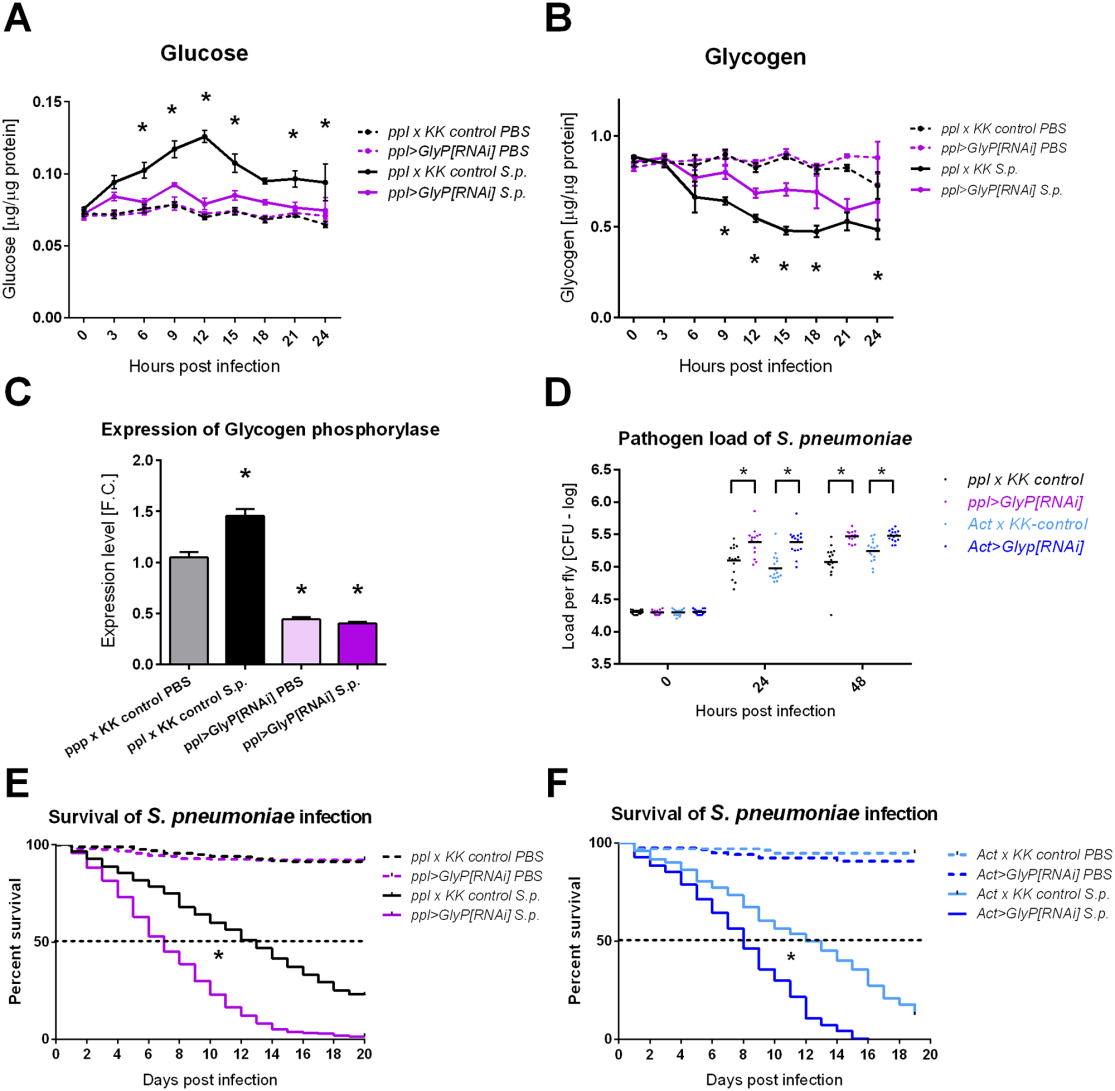
Systemic metabolic switch and glycogen breakdown are required for effective resistance to *Streptococcus pneumoniae*. (A) Free glucose levels in PBS injected (dashed line) and *S*. *pneumoniae* (S.p.) infected flies (solid line) of control (*ppl-Gal4* crossed to *KK* control line labeled as *ppl x KK* control; black) and *ppl>GlyP-RNAi* flies with RNAi induced in fat body (purple). Infection induces hyperglycemia, peaking at 12 hpi, in control flies (significant differences in all 3–24 hpi when compared to uninfected, PBS injected control; not marked). *ppl>GlyP[RNAi]* significantly suppresses this infection-induced hyperglycemia when compared to infected *ppl x KK* control (comparing solid lines marked by asterisks; glucose level is not significantly different in any time point when infected and uninfected *ppl>GlyP RNAi* are compared). (B) Glycogen levels corresponding to measurements in panel A show the decrease of glycogen upon infection (significant differences in 6–24 hpi for *ppl x KK* control and 15–24 hpi for *ppl>GlyP[RNAi]*; not marked). The glycogen decrease is significantly suppressed by *ppl>GlyP[RNAi]*) when compared to infected control (comparing solid lines marked by asterisks). Lines connect mean values with SEM as error bars; significant differences were tested by unpaired t-test (corrected for multiple comparisons using the Holm-Sidak method). (C) Expression of the glycogen phosphorylase increases at 24 hpi with *S*. *pneumoniae* infection in *ppl x KK* control flies. *ppl>GlyP* RNAi (induced in fat body) reduces the whole-fly expression to less than half, thus demonstrating an efficient silencing of glycogen phosphorylase both in uninfected and infected flies. Bars show mean fold change relative to expression of glycogen phosphorylase in uninfected *ppl x KK* control flies obtained by qRT-PCR with SEM as error bars; values were compared with unpaired t-tests and significant differences are marked by asterisks. (D) Comparison of pathogen loads of *S*. *pneumoniae* at 24 and 48 hpi shows that knocking-down glycogen phosphorylase both specifically in the fat body (*ppl>GlyP[RNAi]*); purple) and in the whole flies (*Act>GlyP[RNAi]*; dark blue) significantly increases pathogen loads when compared to appropriate controls (*ppl x KK* control in black and *Act x KK* control in light blue, resp.) Loads, shown in logarithmic scale (values follow lognormal distribution), were compared with unpaired t-tests. (E) and (F) Knocking-down glycogen phosphorylase both specifically in the fat body (*ppl>GlyP[RNAi]*); purple in panel E) and in the whole flies (*Act>GlyP[RNAi]*; dark blue in panel F) significantly reduces survival (P<0.0001) when infected with *S*. *pneumoniae* (solid lines); no differences in survival were detected between genotypes when injected with PBS buffer only (wound control; dashed lines). Survivals were analyzed by both Log-rank and Gehan-Breslow-Wilcoxon tests.

We had previously shown that a systemic metabolic switch, which redirected energy devoted to developmental processes to the immune system, was required for the resistance of *Drosophila* larvae to parasitoid wasp infection [11]. Therefore, we analyzed here the systemic metabolism during *S*. *pneumoniae* infection and found a similar systemic metabolic switch, in which circulating glucose in control flies rose upon infection, peaking at 12 hpi (Fig 2A), while glycogen levels were reduced to half within the first 24 hpi (Fig 2B). In agreement with this glycogen breakdown during infection, the expression of the glycogen phosphorylase rose during *S*. *pneumoniae* infection (Fig 2C).

To test the importance of energy release from glycogen for the immune response, we tested the effect of silencing glycogen phosphorylase (GlyP) during *S*. *pneumoniae* infection. We used Gal4/UAS induced RNA interference (RNAi) with thermosensitive Gal80 to induce RNAi just prior to infection to avoid any developmental effects of silencing GlyP; both control flies and flies with RNAi showed similar levels of glycogen at the beginning of infection (Fig 2B). Knocking down GlyP specifically in the fat body (*ppl>GlyP[RNAi]*; Fig 2C) significantly reduced glycogen breakdown and suppressed hyperglycemia upon infection (Fig 2A and 2B). This led to increased pathogen load (Fig 2D) and decreased survival (Fig 2E). Similar effects on pathogen load and survival were achieved by systemically induced RNAi (*Act>GlyP[RNAi]*) (Fig 2D and 2F).

### Adenosine signaling mediates the systemic metabolic switch

In our previous work, mentioned above [11], we showed that the systemic metabolic switch was mediated by adenosine signaling. Therefore, we used here three different genetic manipulations to investigate the role of adenosine on the immune response against the bacterial infection of adult flies. First, we blocked systemic adenosine signaling by the *adoR* null mutation of the adenosine receptor AdoR. To enhance the effects of adenosine, we lowered an expression of adenosine deaminase ADGF-A first by a heterozygous null mutation in the *ADGF-A* gene (*adgf-a[kar]/+*), since the homozygous mutation causes larval lethality [17]. Second, we lowered the expression of ADGF-A by targeted RNAi in hemocytes (Drosophila immune cells), using Gal4/UAS system [18] when the expression of double stranded RNA for ADGF-A was driven by the hemocyte-specific Hemolectin-Gal4 driver (*Hml>ADGF-A[RNAi]*).

**Fig 3:**
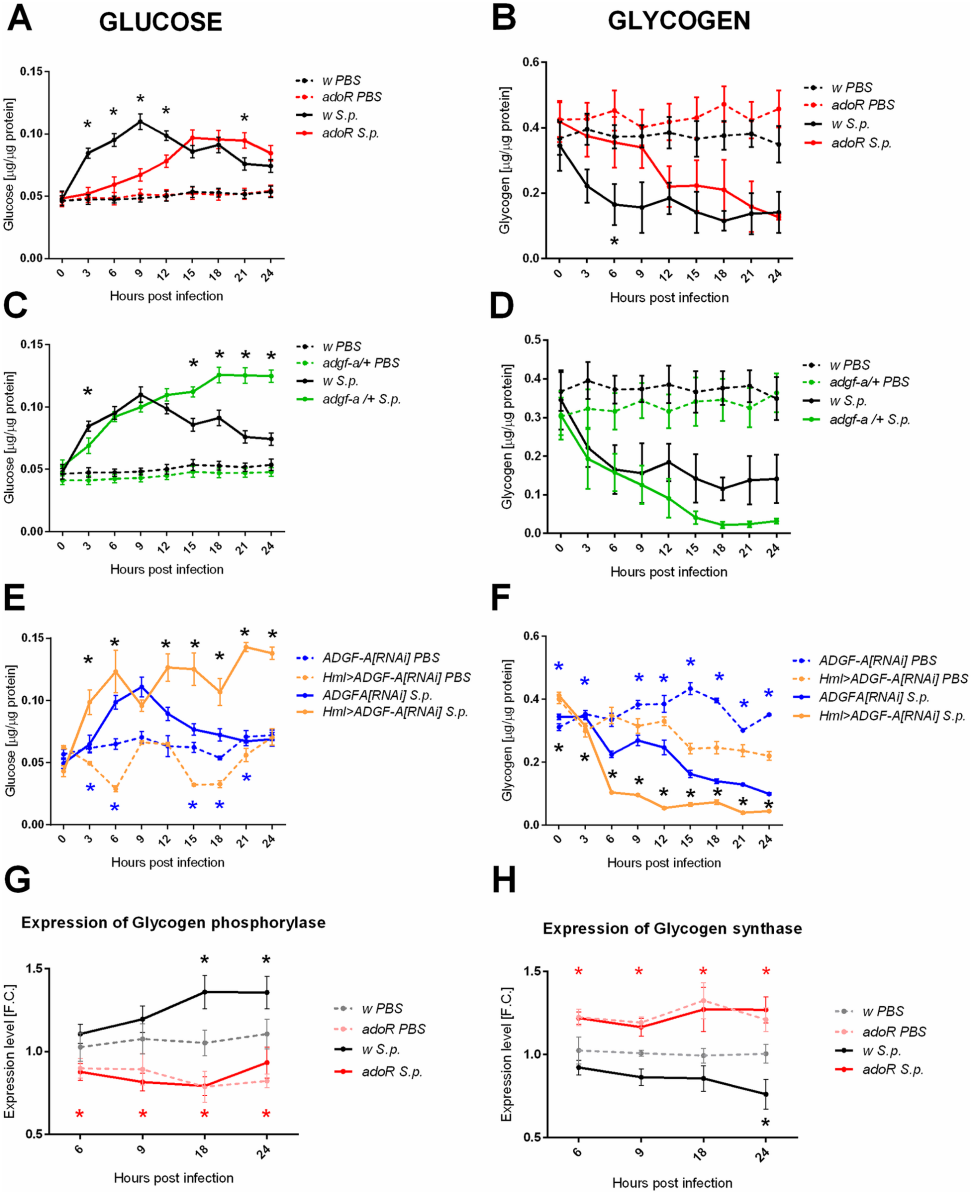
Carbohydrate metabolism during *Streptococcus pneumoniae* infection of flies with manipulated adenosine levels. (A, C and E) Free glucose levels in PBS injected (dashed line) and *S*. *pneumoniae* (S.p.) infected flies (solid line) of different genotypes: *w* control (black), *adoR* (red), and *adgf-a/+* (green) mutants, plus *Hml>ADGF-A[RNAi]* (orange) compared to *ADGF-A[RNAi]* (blue). The *adoR* mutant significantly lowers the early hyperglycemia observed in the *w* control; both *adgf-a/+* and *Hml>ADGF-A[RNAi]* prolongs hyperglycemia from the 9-hpi peak. (B, D and F) Glycogen levels corresponding to the measurements in panels A, C, and E. Although the results are not in all cases statistically significant due to a higher variability in measurement, a trend emerges in which the *adoR* mutant starts to break down glycogen later than the *w* control while glycogen drops lower in both *adgf-a/+* and *Hml>ADGF-A[RNAi]* flies as compared to their respective controls. Lines connecting mean values with SEM as error bars and significant differences (tested by unpaired t-test) marked by asterisks (black asterisks for comparison of S.p. solid lines and blue asterisk for comparison of PBS dashed lines). (G and H) Expression of glycogen phosphorylase increases (panel G) and expression of glycogen synthase decreases (panel H) in *w* control flies during infection (grey and black lines and black asterisks for significant differences). There is no such infection-induced change in *adoR* (pink and red lines) but expression of glycogen phosphorylase is significantly lower and expression of glycogen synthase significantly higher both in uninfected and infected *adoR* when compared to *w* controls (red asterisks). Lines connect mean fold change values of expression relative to expression in uninfected w control at 6 hpi with SEM as error bars; asterisks indicate significant changes (tested by unpaired t-test).

The metabolic switch, induced by *S*. *pneumoniae* infection and expressed by hyperglycemia associated with glycogen breakdown (Fig 2A and 2B), was clearly delayed in the *adoR* mutant, in which the glucose and the glycogen profiles were delayed 6–9 hours compared to the control (Fig 3A and 3B). In contrast, lowering ADGF-A in *adgf-a/+* and *Hml>ADGF-A[RNAi]* flies caused the continuation of hyperglycemia beyond the 9-hpi control peak (Fig 3C and 3E), leading to a deeper depletion of glycogen stores (Fig 3D and 3F), thus causing an expected opposite effect compared to the *adoR* mutation. The role of adenosine signaling in glycogen breakdown is further supported by the expression of the glycogen phosphorylase and glycogen synthase. There was a lower expression of the glycogen phosphorylase and a higher expression of the glycogen synthase in the *adoR* mutant (Fig 3G and 3H). In contrast to the situation in the control flies, where the glycogen phosphorylase rose and the glycogen synthase dropped during infection, their expression did not change in *adoR* (Fig 3G and 3H), supporting the role of adenosine signaling in the regulation of glycogen synthesis/breakdown.

### Adenosine is required for resistance to *S*. *pneumoniae*

**Fig 4:**
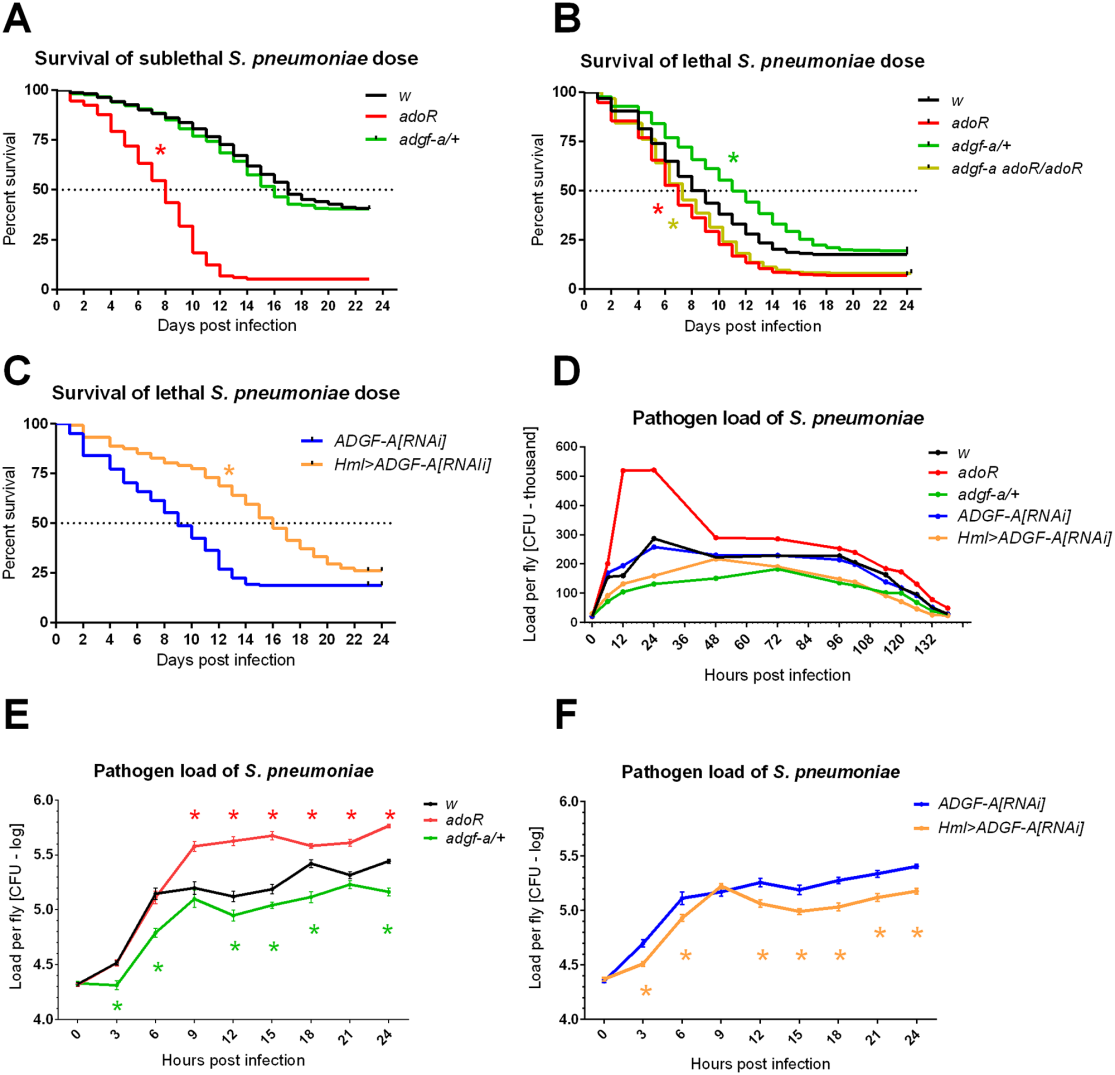
Host survival and pathogen loads during *Streptococcus pneumoniae* infection of flies with manipulated adenosine levels. (A) The *adoR* mutation (red) significantly reduces survival (P<0.0001) while *adgf-a/+* (green) is comparable (P = 0.1647) to the *w* control (black) when infected with a sub-lethal dose of *S*. *pneumoniae*. (B and C) Both the *adgf-a/+* heterozygous mutant (green) and *Hml>ADGF-A[RNAi]* (orange) flies increases survival (both P<0.0001) compared to *w* and *ADGF-A[RNAi]* (blue) controls when infected with a lethal dose of *S*. *pneumoniae*. The effect of *adgf-a/+* is fully suppressed (P<0.0001; purple star) by the *adoR* mutation when double *adgf-a adoR/adoR* mutant (olive) shows similar decrease to *adoR* (P = 0.9222 when *adgf-a adoR/adoR* compared to *adoR*). (D) Pathogen loads of *S*. *pneumoniae* in colony forming units (CFU) per fly were determined over a span of 6 days and are shown as lines connecting the mean values in linear scale (multiplied by thousand; see S1 Fig for the corresponding dot plots). (E) and (F) Pathogen loads within the first 24 hours post-infection in logarithmic scale (values follow lognormal distribution) with significant differences marked by asterisks (color determines the compared genotype to control, i.e. either *w* or *ADGF-A[RNAi])*; *adoR* increases while both *adgf-a/+* and *Hml>ADGF-A[RNAi]* decreases the pathogen loads (lines connect the mean values with SEM as error bars; see also S2 Fig for appropriate dot plots). Survival was analyzed by Log-rank and Gehan-Breslow-Wilcoxon tests and pathogen loads with unpaired t-tests (corrected for multiple comparisons using the Holm-Sidak method).

Since the immune response was dependent on the systemic metabolic switch and the results above showed that the switch was mediated by adenosine, we tested the resistance of the flies with manipulated adenosine signaling. Injecting a sublethal dose (15 000 CFUs or less) allowed most of the control flies to survive the acute phase of infection (the first 7–8 days; Fig 4A) while a lethal dose (20 000 CFUs or more) killed 50% of control flies by day 8 and led to less than 25% survival overall (Fig 4B). The *adoR* mutation decreased survival compared to the *w* control when injected with both sublethal and lethal doses (Fig 4A and 4B). While the lethal dose killed most of the control flies, *adgf-a/+* and *Hml>ADGF-A[RNAi]* flies improved survival during the acute phase (Fig 4B and 4C). The observed effects were not caused by a difference in vigor [19] among these genotypes since the injection of PBS buffer led to comparable survival under the experimental conditions (Fig 1D).

Fig 4D shows that *S*. *pneumoniae* load increased mostly within the first 24 hpi, reaching up to three hundred thousand CFUs per fly, and was eventually cleared within 5–6 days. We monitored the pathogen load during this 6-day span and detected differences among the tested genotypes, especially during the first 24–48 hpi (Fig 4D) when most flies were still alive (Fig 4A–4C). At later time points, differences in pathogen load in flies, which were still alive and available for pathogen load measurements, virtually disappeared between genotypes (Fig 4D and S1 Fig). Therefore, we monitored in detail the growth of *S*. *pneumoniae* during the first 24 hpi. In agreement with the relative survival rates, the number of CFUs was significantly higher in *adoR* and lower in both *adgf-a/+* and *Hml>ADGF-A[RNAi]* compared to the controls (Fig 4E and 4F and S2 Fig). Although the *adgf-a/+* and *Hml>ADGF-A[RNAi]* flies had lower pathogen loads and better survived the acute phase of *S*. *pneumoniae* infection, many of them died after the acute phase and thus their overall survival was eventually comparable to the control (Fig 4B and 4C). These results suggest that *adgf-a/+* and *Hml>ADGF-A[RNAi]* better controlled the pathogen load, leading to their better survival of the acute infection phase, but eventually have problems with recovery from infection; the reason for the recovery problem is unknown.

It is important to stress that the use of the heterozygous *adgf-a* knock out mutant as a model led to a similar result as that obtained by depleting ADGF-A in hemocytes by RNAi (*Hml>ADGF-A[RNAi])* and to an opposite result to that obtained with the *adoR* mutation, thus connecting the phenotypes specifically to the genetic manipulations of extracellular adenosine. We were not able to detect clear and reproducible changes in the extracellular adenosine levels, having problems rapidly collecting a reasonable amount of hemolymph from adult flies and due to the short half-life of this molecule upon sample collection. Therefore, to determine whether the observed effects of lowering ADGF-A expression are indeed due to adenosine signaling, we tested resistance to *S*. *pneumoniae* in a double mutant, heterozygous for *adgf-a* and homozygous for *adoR* (*adgf-a adoR/adoR*), lowering ADGF-A expression and at the same time removing adenosine signaling. Fig 4B and S3 Fig demonstrate that the effect of *adgf-a/+* is completely suppressed by the *adoR* mutation in the *adgf-a adoR/adoR* double mutant when both survival and pathogen loads are similar to the *adoR* single mutant. This demonstrates that the observed effects in *adgf-a/+* are dependent on adenosine signaling.

**Fig 5:**
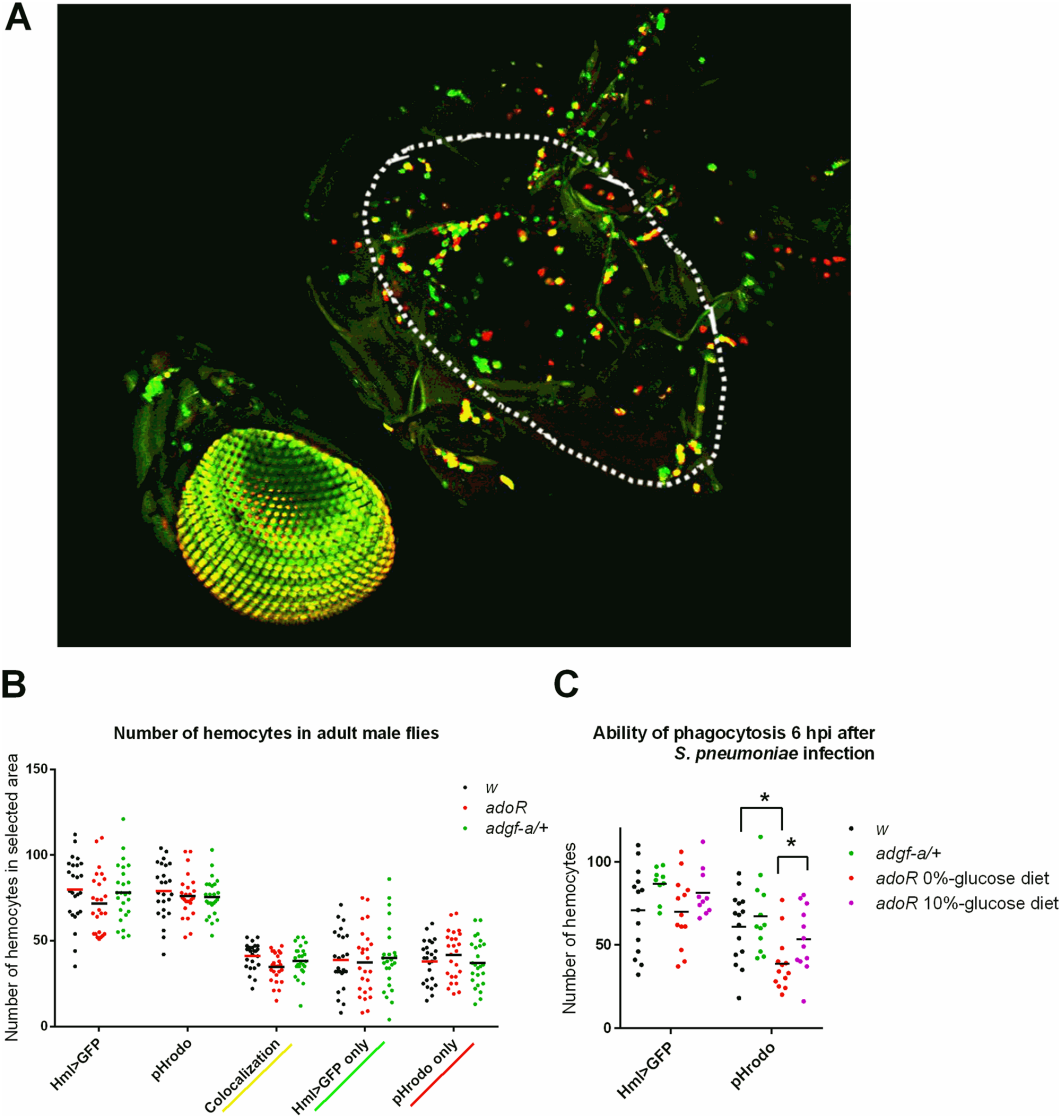
Adult hemocytes quantification and phagocytosis capability. (A) Confocal microscopy image of a fly with genetically labeled Hml>GFP hemocytes (green) and injected with pHrodo, which fluoresces red after being phagocytosed; yellow coloration marks co-localization of green Hml>GFP positive cells being able to phagocytose the red pHrodo. White line labels the area of side thorax were hemocytes were counted. (B) Number of Hml>GFP positive cells and pHrodo phagocytosing cells (and their combinations) in *w* control (black) and the *adoR* (red) and *adgf-a/+* (green) mutant flies. Cells were counted in the area marked in (A). Neither mutant genotype affects the total number of hemocytes or the basal capacity of phagocytosis. (C) Number of Hml>GFP and pHrodo-positive cells in flies infected by *S*. *pneumoniae* 6 hours prior to pHrodo injection. While there is comparable number of Hml>GFP cells upon infection in all genotypes, the infected *adoR* mutant shows a lower number of phagocytic cells with a carbohydrate-poor, 0%-glucose diet, which is improved by a carbohydrate-rich, 10%-glucose diet (purple). The number of cells was counted in the same area as depicted in (A). Dots in graphs show individual counts, with lines marking the mean values; asterisks mark significantly different values determined by t-tests.

As shown above, resistance to *S*. *pneumoniae* is dependent on phagocytosis; blocking phagocytosis with latex beads erased differences between genotypes (Fig 1B and 1C) demonstrating that the differences are connected to phagocytosis. The effectivity of the phagocytic response could be influenced by the number of hemocytes, which in turn is dependent on genetic background [20]. A comparable number of active phagocytes, and even adult hemocytes in general, among *w*, *adoR and adgf-a/+* genotypes (with unified genetic backgrounds) showed that it was not the different number of phagocytes (Fig 5A and 5B) causing the observed effects of these genotypes on resistance to *S*. *pneumoniae*. We detected a significantly lower number of cells labeled by pHrodo, a marker of active phagocytosis, in the *adoR* mutant challenged by *S*. *pneumoniae* 6 hours prior to a pHrodo injection when the total number of the Hml>GFP-labeled hemocytes was the same as in the control (Fig 5C). This lower phagocytosis in challenged *adoR* was significantly increased by a carbohydrate-rich, 10%-glucose diet (Fig 5C). So, although there are comparable numbers of phagocytes with comparable basal phagocytic capacity (without challenge), their activity (but not their numbers) is lower in the *adoR* mutant when the immune response is activated by the challenge.

### Role of adenosine signaling during *Listeria monocytogenes* infection

**Fig 6:**
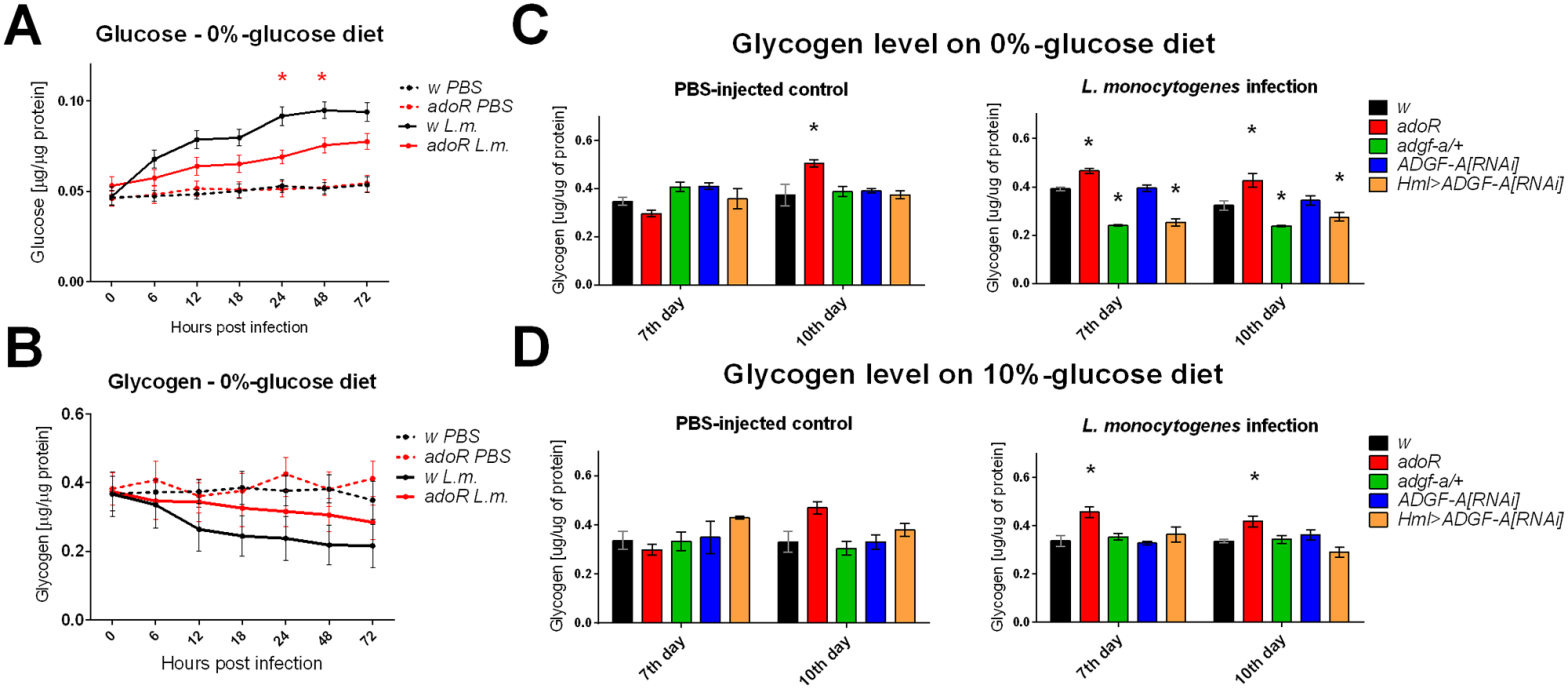
Carbohydrate metabolism during *Listeria monocytogenes* infection. (A) Free glucose levels in PBS injected (dashed line) and *L*. *monocytogenes* (L.m.) infected flies (solid line) of *w* control (black) and the *adoR* mutant (red) on a carbohydrate-poor, 0%-glucose diet. *adoR* lowers hyperglycemia observed in the *w* control. (B) Glycogen levels corresponding to measurements in (A); although the results are not statistically significant due to variability in measurement, the *adoR* mutant does not break down as much glycogen as the *w* control, which corresponds to glucose measurements. Lines connect mean values with SEM as error bars; significant differences (tested by unpaired t-test) marked by asterisks. (C, D) Glycogen levels in PBS-injected flies (left) and during the chronic phase of *L*. *monocytogenes* infection (right) measured at day 7 and 10 post infection on flies reared on carbohydrate-poor, 0%-glucose (C) and carbohydrate-rich, 10%-glucose (D) diets. Significantly lower glycogen levels in *adgf-a/+* and *Hml>ADGF-A[RNAi]* flies during *L*. *monocytogenes* infection (C) are rescued by the carbohydrate-rich diet (D). Columns show mean values with SEM as error bars and asterisks mark significant difference (tested by unpaired t-test) to control (*w* for *adoR* and *adgf-a/+*, respectively, and *ADGF-A[RNAi]* for *Hml>ADGF-A[RNAi]*).

Next, we tested a different type of infection, triggered by *Listeria monocytogenes*, which causes a chronic intracellular infection leading eventually to the death of the host [21]. Intracellular infection is established by phagocytosis followed by the escape of *L*. *monocytogenes* from the phagosome to the cytoplasm. Similarly to *S*. *pneumoniae* infection, the *L*. *monocytogenes* infection also led to a systemic metabolic switch, characterized by hyperglycemia (Fig 6A); glycogen stores were accordingly reduced (Fig 6B). The *adoR* mutation reduced hyperglycemia (Fig 6A) and increased glycogen storage compared to the control (Fig 6B), the two effects persisting even during the chronic phase (Fig 6C). Since there was no peak of hyperglycemia during the acute phase of *L*. *monocytogenes* infection, we did not detect a difference in glucose level in the *adgf-a/+* flies (S4 Fig), however both the *adgf-a/+* and *Hml>ADGF-A[RNAi]* flies had significantly lower glycogen stores during the chronic phase (Fig 6C).

**Fig 7:**
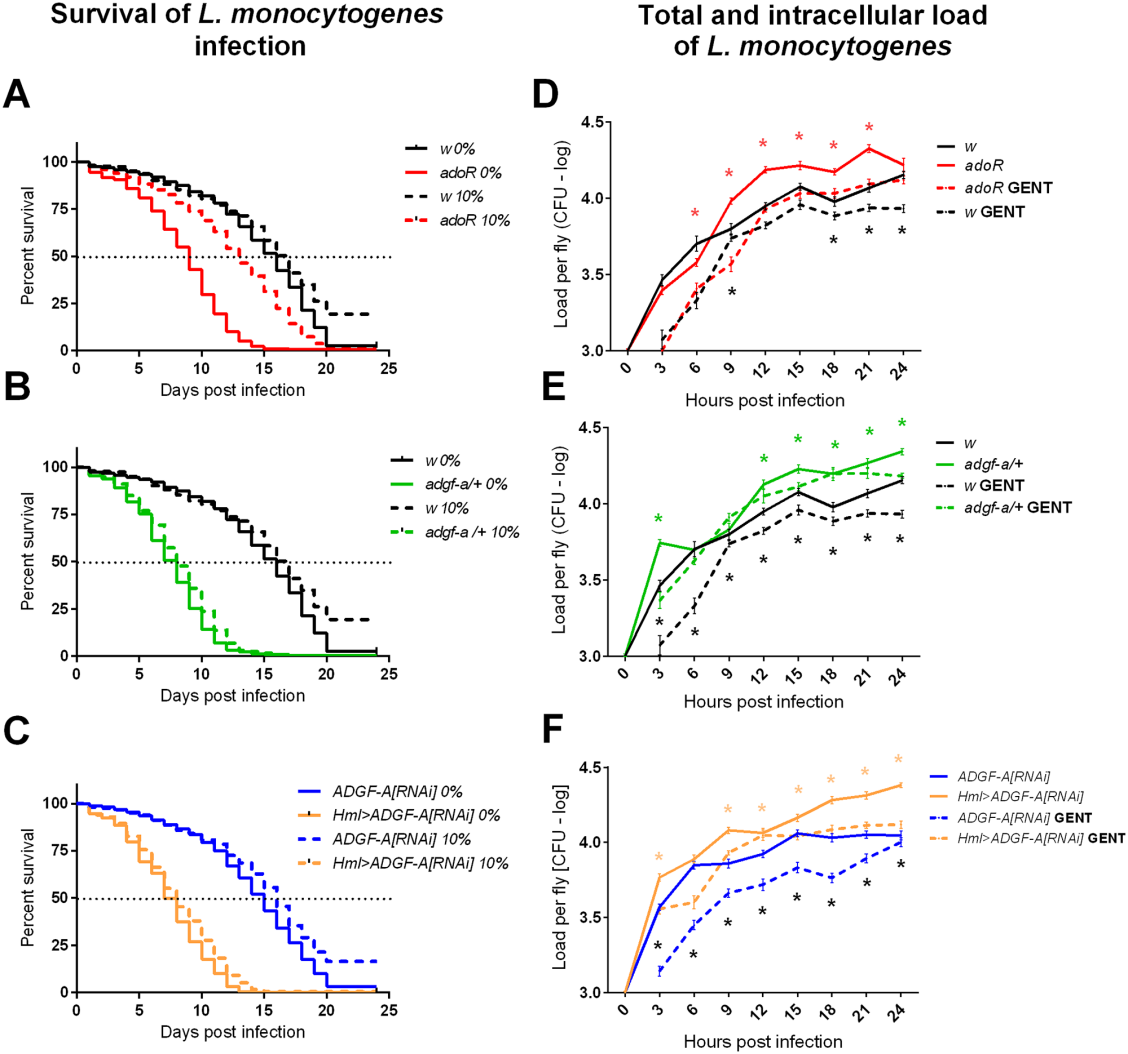
Host survival and pathogen load during *Listeria monocytogenes* infection. (A-C) Survival upon *L*. *monocytogenes* infection was analyzed on flies reared on carbohydrate-poor, 0%-glucose (marked as 0%; solid lines) and carbohydrate-rich, 10%-glucose (marked as 10%; dashed lines) diets and were analyzed by both Log-rank and Gehan-Breslow-Wilcoxon tests. (A) The *adoR* mutation (red) significantly reduces the survival (P<0.0001) of flies on the 0%-glucose diet compared to the *w* control. Survival of the *adoR* mutant is significantly improved by a 10%-glucose diet, which does not influence the survival of control flies but shifts the median time to death of *adoR* from 9 to 13 days (P<0.0001). (B) The *adgf-a/+* mutation (green) significantly reduces survival (P<0.0001) on both diets. (C) The *Hml>ADGF-A[RNAi]* (orange) flies also significantly reduce survival (P<0.0001) compared to *ADGF-A[RNAi]* (blue) control on both diets. (D-F) Pathogen load of *L*. *monocytogenes* in CFU per fly in logarithmic scale (values follow lognormal distribution) within the first 24 hpi. Lines connect mean values with SEM as error bars; solid lines show the total number of bacteria and dashed lines show the number of intracellular bacteria after the gentamycin treatment killing the extracellular population (marked as GENT). Black asterisks mark significant differences between intracellular bacteria (comparing dashed lines), colored asterisk mark significant differences between total bacteria (comparing solid lines); results were compared by unpaired t-tests corrected for multiple comparisons using the Holm-Sidak method. Statistics and detailed dot plot graphs can be found in S5 Fig. (D) The *adoR* mutant increases the total number of bacteria, mainly due to the extracellular population. Both *adgf-a/+* (E) and *Hml>ADGF-A[RNAi]* (F) also increase the total pathogen load, which is mostly due to the intracellular bacteria because the numbers after gentamicin treatment (dashed lines) are markedly increased compared to controls.

The length of host survival depends on the injected dose [19] and the genetic background that determines, for example, the number of phagocytes [20]. We injected 1000 bacteria (S5A Fig) leading to a median time to death of 17 days in our control flies (Fig 7A). Both the *adoR* mutation and lowering ADGF-A by *adgf-a/+* and *Hml>ADGF-A[RNAi]* shortened survival during *L*. *monocytogenes* infection to a median time to death of only 8 days (Fig 7A–7C). The observed shorter survival durations were associated with increased pathogen load (Fig 7D–7F), ostensibly suggesting a lower resistance to *L*. *monocytogenes* for all three genotypes. However, distinguishing the intracellular and extracellular *L*. *monocytogenes* populations revealed a more complicated picture. We used gentamycin, an antibiotic that is unable to cross cellular membranes, to determine the total number of bacteria (without gentamycin treatment) and the number of intracellular bacteria (after gentamycin treatment). This gentamycin-chase assay described in [5] showed that the increased pathogen load in the *adoR* mutant was mainly due to an increased extracellular population (Fig 7D and S5 Fig). On the other hand, the increased pathogen load in *adgf-a/+* and *Hml>ADGF-A[RNAi]* flies was almost solely caused by an increase in the intracellular *L*. *monocytogenes* population (Fig 7E and 7F and S5A Fig); the extracellular population disappeared faster in *adgf-a/+* and *Hml>ADGF-A[RNAi]* flies compared to the control suggesting more effective phagocytosis upon lowering ADGF-A.

**Fig 8:**
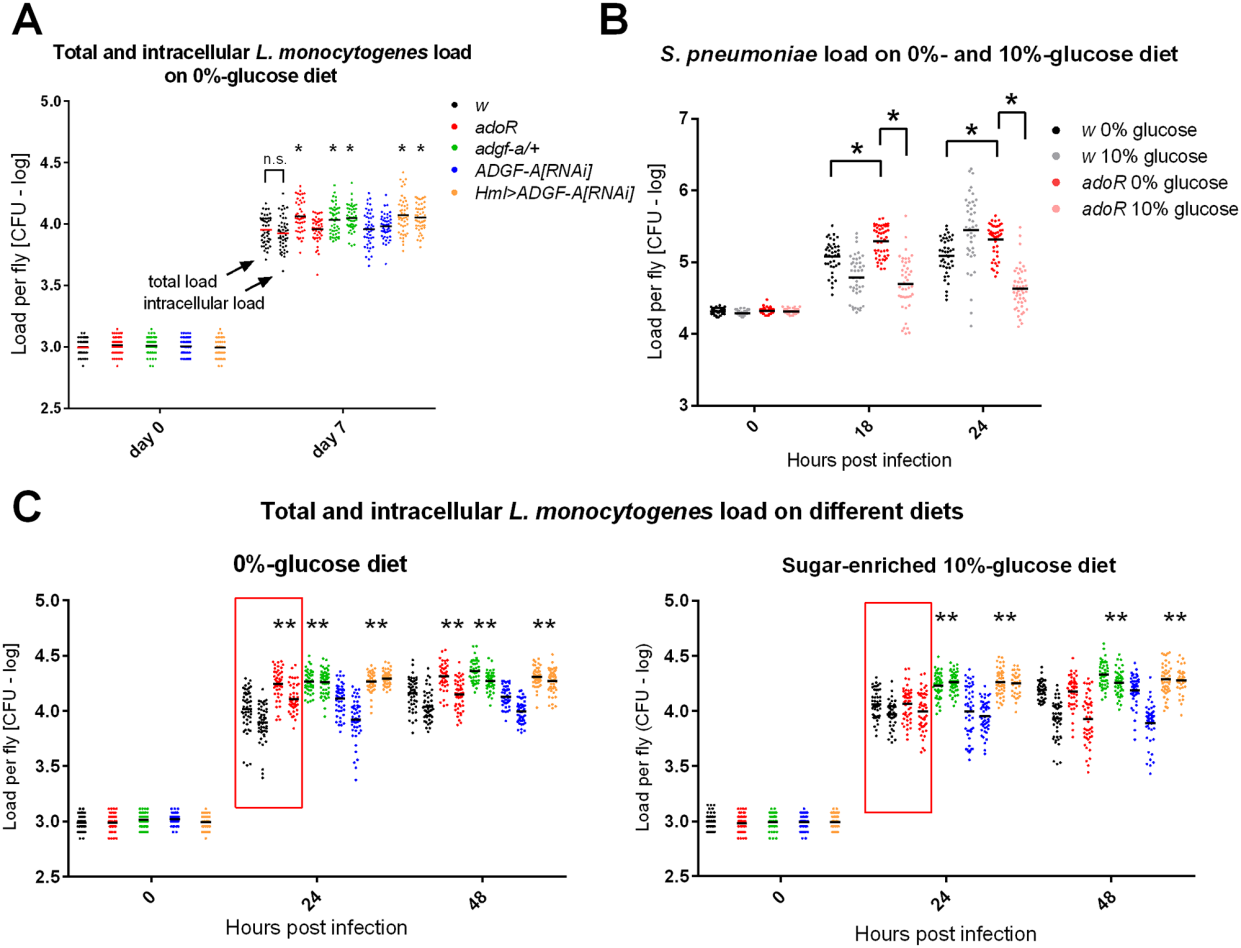
Pathogen loads on carbohydrate-poor and carbohydrate-rich diets. (A) Pathogen load of *L*. *monocytogenes* in CFU per fly on a carbohydrate-poor, 0%-glucose diet in logarithmic scale during the chronic phase (at day 7 post infection). Each color represents a different genotype according to the legend (the same for panel C); total number of bacteria is on left and the number of intracellular bacteria after the gentamycin treatment is on right for each genotype. The two numbers for *w* control are the same (labeled as n.s.) demonstrating that all bacteria are intracellular at this stage. *adoR* increases the total number of bacteria due to the extracellular population while both *adgf-a/+* and *Hml>ADGF-A[RNAi]* exhibit increases in intracellular numbers. Asterisks label statistically significant differences (tested by unpaired t-test) when comparing the load of total or intracellular bacteria in a particular genotype to its counterpart in the control (*adoR* and *adgf-a/+* to *w* and *Hml>ADGF-A[RNAi]* to *ADGF-A[RNAi]*). (B) Pathogen load of *S*. *pneumoniae* increases in *adoR* compared to *w* control on a carbohydrate-poor diet, an effect that is compensated for by a carbohydrate-rich diet. (C) Total and intracellular *L*. *monocytogenes* loads during the first two days of infection of flies on a carbohydrate-poor, 0%-glucose (left) and a carbohydrate-rich, 10%-glucose (right) diets; labelled as in (A). Red squares highlight the effect of a sugar-enriched diet, which suppresses the increased pathogen load in *adoR* on sugar-poor diet.

The increased intracellular pathogen load in *adgf-a/+* and *Hml>ADGF-A[RNAi]* flies persisted even during the chronic phase (day 7, Fig 8A), when the phagocytosis likely no longer played a role because all bacteria were intracellular (*i*.*e*. no difference in total and intracellular load in *w* control in Fig 8A). The *adoR* mutation also increased the total pathogen load at day 7, although this represented a surge in the extracellular population, as the intracellular population was similar to the control (Fig 8A). Melanization was previously shown to be important in controlling extracellular *L*. *monocytogenes* loads when lowering the melanization response led to an increased extracellular bacteria population [22]. The increased presence of extracellular bacteria in *adoR* flies most likely stimulated an increase in disseminated melanization [22] at day 7 (S6 Fig), when only 37% of *w* showed melanization (with extensive melanization in 12% of flies; n = 40) compared to 63% of *adoR* flies (with extensive melanization in 30% fies; n = 24; S6 Fig).

### Sugar-enriched diet rescues the *adoR* mutant resistance defect

In our previous work, we had been able to partially rescue the effect of *adoR* on the metabolic switch and immune response by providing more glucose in the fly diet [11]. Here we show that a sugar-enriched, 10%-glucose diet significantly increased the survival of *adoR* flies (Fig 7A and S7 Fig) and lowered the pathogen load to control levels during both *S*. *pneumoniae* and *L*. *monocytogenes* infections (Fig 8B and 8C). The sugar-enriched diet did not influence the survival or pathogen load of *adgf-a/+* or *Hml>ADGF-A[RNAi]* flies (Figs 7B, 7C and 8C).

### Expression of antimicrobial peptides during *S*. *pneumoniae* and *L*. *monocytogenes* infection

The results above demonstrated the effects of adenosine manipulation on immune defenses associated with phagocytosis and metabolism. Host immunity may also be mediated by the production of antimicrobial peptides (AMPs) and, therefore, we analyzed the expression of four selected AMPs (S8 Fig). Both *S*. *pneumoniae* and *L*. *monocytogenes* induced the expression of Defensin, Diptericin, Drosocin and Metchnikowin, albeit to different extents. As shown in S8 Fig, during *S*. *pneumoniae* infection, the *adoR* mutant showed a lower expression of Defensin, Diptericin, and Drosocin at 6 hpi but not at 18 hpi. In the case of *L*. *monocytogenes* infection, only the Metchnikowin expression was strongly reduced at 6 hpi in the *adoR* mutant. The lower expression of three out of four AMPs in *adgf-a/+* or *Hml>ADGF-A[RNAi]* flies infected with both *S*. *pneumoniae* and *L*. *monocytogenes* (S8 Fig) rather reflects the lower extracellular-bacteria load in these flies than being the reason for more effective clearance of this extracellular population.

### ADGF-A expression

**Fig 9:**
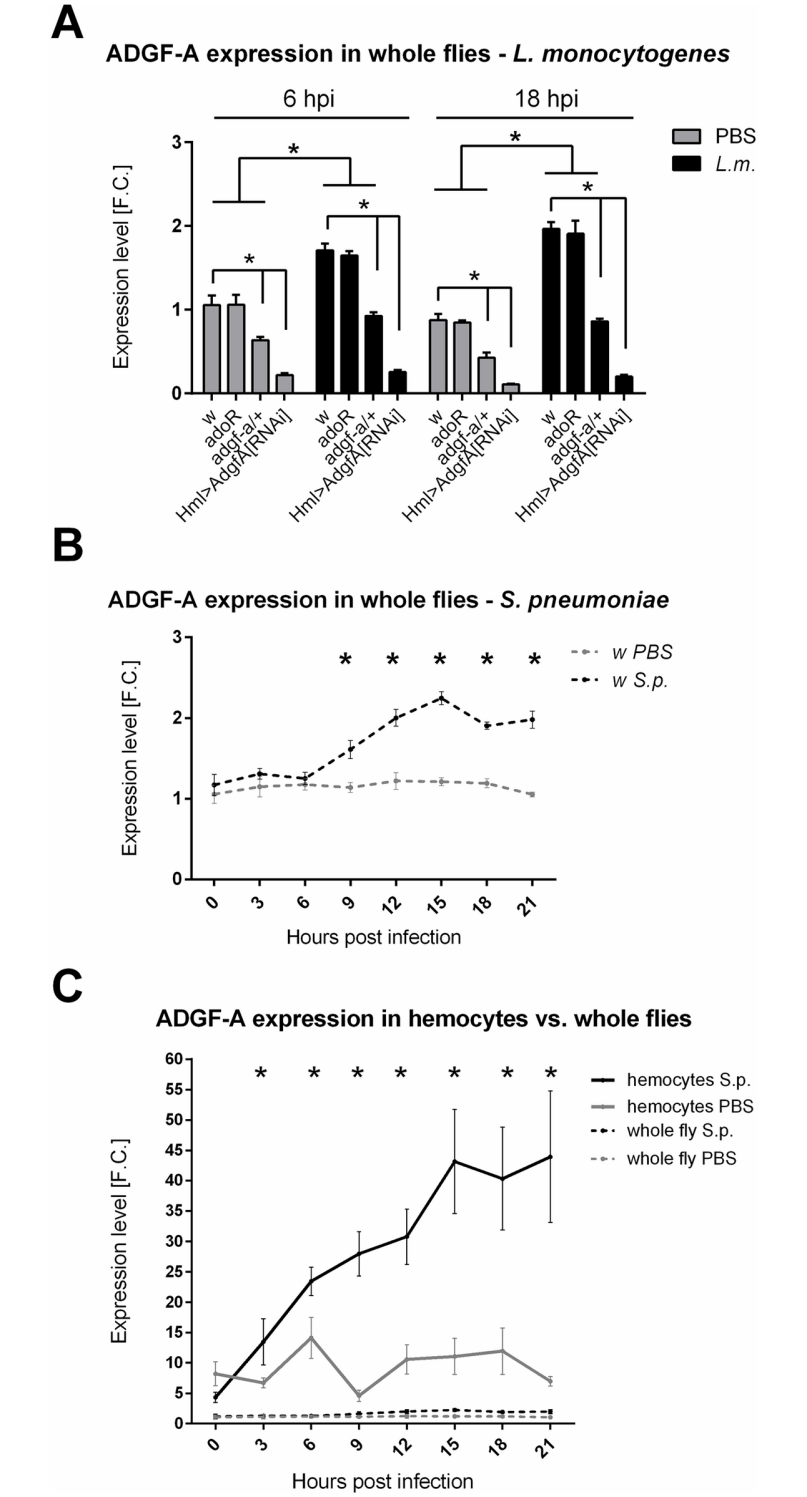
ADGF-A expression during infection. (A) Expression of ADGF-A in PBS-injected (grey bars) and flies infected by *L*. *monocytogenes* (*L*.*m*., black bars) 6 and 18 hpi demonstrates a significant increase in expression caused by infection in all *w* control, *adoR* and *adgf-a/+* genotypes. *Hml>ADGF-A[RNAi]*) shows an effective silencing of ADGF-A expression to less than 20% under all circumstances. Bars show mean fold change values of expression relative to expression in uninfected w control at 6 hpi with SEM as error bars; asterisks indicate significant changes tested by unpaired t-test. (B) Time-course analysis of ADGF-A expression in whole flies upon infection with *S*. *pneumoniae* (S.p.), showing a significant increase in its expression from 9 hpi and reaching two-fold increase. Lines connect mean fold change values of expression relative to expression in uninfected w control at time 0 with SEM as error bars. (C) Time-course analysis of ADGF-A expression in hemocytes showing one order of magnitude higher expression compared to the expression at the whole fly level. Hemocytes also increase ADGF-A expression during infection reaching 5-fold increase. Lines connect mean fold change values of expression relative to whole-fly expression in uninfected *w* control at time 0 with SEM as error bars. Asterisks indicate significant changes (tested by unpaired t-test and corrected for multiple comparisons using the Holm-Sidak method in B and C).

Adenosine is regulated by the adenosine deaminase ADGF-A [17]. Gene expression analysis showed that *ADGF-A* expression increased both upon *L*. *monocytogenes* and *S*. *pneumoniae* challenges (Fig 9A and 9B). Fig 9A also demonstrates that there is lower *ADGF-A* mRNA expressed in the *adgf-a/+* heterozygous mutant; some of this mRNA might possess a premature stop codon (*adgf-a* mutation) leading to aberrant protein production, which is not distinguishable by the used q-PCR (for a description of this mutation, see [23]). The Hml-induced RNA interference of ADGF-A specifically in hemocytes effectively silenced the ADGF-A expression, demonstrating the functionality of the Hml>ADGF-A[RNAi] knockdown (Fig 9A). It is important to note that the expression was measured on the whole-organism level, suggesting that the expression of ADGF-A in hemocytes, where Hml-Gal4 driver is expressed, represents a majority of the whole organism expression of this gene. A hemocyte-specific expression analysis (Fig 9C) confirmed this speculation when hemocytes showed a one order of magnitude higher expression of ADGF-A compared to the expression measured in whole flies and this expression rose four times during infection demonstrating that hemocytes are the primary producers of ADGF-A. The time-course expression during *S*. *pneumoniae* infection (Fig 9B and 9C) also showed that the ADGF-A expression rose after 9 hpi, which coincided with the down-regulation of hyperglycemia during *S*. *pneumoniae* infection (Fig 3A).

## Discussion

In this work, we used two types of bacterial infection of adult *D*. *melanogaster*, one caused by the facultative intracellular bacterium *L*. *monocytogenes* and the other triggered by the extracellular pathogen *S*. *pneumoniae*, to test the effects of the genetic manipulation of adenosine signaling on host-pathogen interactions. To block systemic adenosine signaling, we used a null *adoR* mutation in the adenosine receptor and to enhance the effects of adenosine, we either removed one copy of the adenosine deaminase ADGF-A gene or lowered its expression by RNAi. We show that both infections are associated with a systemic metabolic switch manifested by hyperglycemia at the expanse of the glycogen stores. Manipulating e-Ado signaling influences this metabolic switch and, at the same time, affects host resistance. The activated immune system requires an increased supply of energy/nutrients [24]. Therefore, we propose that the observed e-Ado-mediated systemic metabolic switch supplies the immune system with the required nutrition, and thus is important for the effectivity of the immune response.

Resistance to *S*. *pneumoniae* was shown to be dependent on effective phagocytosis by hemocytes [16]. We confirm here the crucial role of phagocytosis by injecting latex beads which jam hemocytes, making the hemocytes unable to phagocytose. Blocking the phagocytosis made flies extremely sensitive to *S*. *pneumoniae* infection and eliminated the differences in responses between the control and mutants used in this work. The observed effects of Ado manipulation on immunity are not due to an altered phagocyte number [20] since all the strains used in this work have comparable numbers of hemocytes, including active phagocytes.

We show here that *S*. *pneumoniae* infection is associated with systemic metabolic switch manifested by hyperglycemia (peaking at 9–12 hpi) at the expanse of the glycogen stores, when glycogen drops to less than one third within 24 hpi. This could merely be a pathological consequence of the infection, however phagocytic cells are known to increase glycolysis and glucose consumption [25] and thus the systemic metabolic switch may also be a reflection of an increased need for energy by the hemocytes phagocytosing *S*. *pneumoniae*. The latter possibility is strongly supported by our experiment with knocking down GlyP, an enzyme responsible for glycogen breakdown. Knocking down GlyP in the fat body immediately prior to infection decreased resistance to *S*. *pneumoniae*, demonstrating that the glucose, liberated from the glycogen stores, is required for effective phagocytosis. Therefore, the observed systemic metabolic switch is most likely an active process ensuring adequate energy supply to the activated immune system.

Our previous work [11] demonstrated that the immune cells of Drosophila larva release Ado during parasitoid wasp infection to mediate a systemic metabolic switch and thus to supply the immune system with the required nutrients. Here we show that blocking adenosine signaling by *adoR* mutation prevents the metabolic switch during *L*. *monocytogenes* infection and postpones the hyperglycemic peak and glycogen use during *S*. *pneumoniae* infection. This notion is further supported by transcriptional data showing that the glycogen synthesis/breakdown is under the AdoR control. Furthermore, hyperglycemia during *S*. *pneumoniae* infection peaks at 9 hpi, which coincides with a rise in ADGF-A expression. When ADGF-A action is lowered by the heterozygous *adgf-a* mutation or RNAi, hyperglycemia continues at the increased expense of the glycogen stores. All these results together demonstrate that the systemic carbohydrate metabolism is regulated by e-Ado in adult flies during infection. The AdoR signaling causes a release of energy, *i*.*e*. hyperglycemia associated with decreased glycogen stores and the e-Ado-mediated release of energy is regulated by ADGF-A.

The lower resistance of the *adoR* mutant to *S*. *pneumoniae* is then in agreement with the role of Ado signaling in the systemic metabolic switch and with the importance of this switch for the effective immune response. Therefore, we propose that adenosine signaling mediates the supply of energy to the activated immune system. This is supported by our experiments with a carbohydrate-rich diet compensating for the missing switch in systemic metabolism in the *adoR* mutant when this diet increases phagocytosis, normalizes the pathogen load and ultimately increases survival in the *adoR* mutant when compared to the carbohydrate-poor diet. These observations are similar to our previous work showing that a carbohydrate-rich diet rescued the effect of *adoR* on the production of immune cells during parasitoid wasp infection [11]. The role of e-Ado in supplying the energy to immune cells is further supported by the opposite effect achieved by removing one copy of ADGF-A or lowering its expression by RNAi. These genetic manipulations have the opposite effect on carbohydrate metabolism compared to *adoR* and at the same time lower the pathogen load during *S*. *pneumoniae* infection, demonstrating more effective phagocytosis in these flies. The complete suppression of this increased resistance by simultaneously mutating *adoR* demonstrates that the effect of lowering ADGF-A is indeed due to adenosine signaling. We can then conclude that e-Ado mediates a systemic metabolic switch which is important for supplying energy to immune cells, and e-Ado is thus crucial for effective phagocytosis and host resistance to *S*. *pneumoniae*.

*S*. *pneumoniae* infection provides a simpler model of host-pathogen interaction in which the clearance is crucially dependent on phagocytosis and the host either clears the bacteria or the pathogen outgrows and kills the host. The other pathogen, *L*. *monocytogenes*, is a facultative intracellular bacterium causing a chronic and ultimately lethal infection in flies. Phagocytosis is actually a way for this pathogen to colonize the host [21]; the intracellular population is eventually established following escape from the phagosome. Infection by this pathogen causes a systemic metabolic switch similar to the one caused by *S*. *pneumoniae*, i.e. hyperglycemia associated with the loss of glycogen stores. Here, as is the case in *S*. *pneumoniae*, adenosine mediates this switch because *adoR* decreases the infection induced-hyperglycemia associated with a lower loss of glycogen while lowering ADGF-A leads to a greater loss of glycogen.

The *adoR* mutation increases the extracellular load of *L*. *monocytogenes* suggesting that, similarly to the case of *S*. *pneumoniae* infection, this mutation decreases the effectivity of phagocytosis via the suppression of the adenosine-mediated metabolic switch. This is supported by evidence of rescue with an increase of glucose in the diet, which normalizes pathogen load and increases the survival of the *adoR* mutant. In agreement with this, lowering ADGF-A leads to the opposite effect, in which this manipulation leads to a faster clearance of the extracellular *L*. *monocytogenes* population associated with the increased intracellular population, suggesting more effective phagocytosis. Thus far, the results obtained with *L*. *monocytogenes*, focused on the metabolism and early response associated with phagocytosis, are similar to those obtained with *S*. *pneumoniae*. Phagocytosis is not, of course, the only defense mechanism available to the flies, two other mechanisms, melanization and antimicrobial peptides, are discussed further.

Unlike to *S*. *pneumoniae* infection, both blocked and enhanced Ado signaling decreased the survival of flies upon *L*. *monocytogenes* infection. This decreased survival is associated with increased total pathogen loads in all examined *adoR*, *adgf-a/+*, and *Hml>ADGF-A[RNAi]* flies. In the *adoR* mutant, the larger pathogen load is mainly due to an increased extracellular population, as mentioned above. In contrast, the increased pathogen load in *adgf-a/+* and *Hml>ADGF-A[RNAi]* flies is almost completely caused by an increase in the intracellular *L*. *monocytogenes* population. The increased intracellular load can be due to less effective intracellular defense mechanisms, which remains mostly unexplored in flies [26], or due to an increased carrying capacity for the pathogen at the expanse of the host energy reserves as suggested by a greater loss of glycogen reserves in flies with lowered ADGF-A. An increased bacteria population obviously requires more nutrients. Among the important virulence factors of *L*. *monocytogenes* is the bacterial homolog of glucose- 6-phosphate translocase, which allows the pathogen to exploit hexose phosphates from the host cell as a carbon source [27]. In addition, *L*. *monocytogenes* hijacks host cell actin polymerization for its propagation [28]. Since this process requires energy, an increased supply to an infected cell may potentially further promote the propagation of this intracellular pathogen. The question is whether the nutrient supply to infected cells is the limiting factor for pathogen proliferation and propagation, however, there is evidence that the proliferation capacity of intracellular pathogens is strongly influenced by host metabolism [4]. If so, as a reaction to the increased release of energy from the host stores caused by lowering ADGF-A, the carrying capacity could increase, and thus could lead to the observed increase in the intracellular pathogen load. However, we cannot exclude the possibility that lowering ADGF-A somehow decreases the host intracellular defense and the associated wasting is just a consequence of increased pathogen load.

Carbohydrate-rich diet normalizes the pathogen load in the *adoR* mutant, decreasing the extracellular population of *L*. *monocytogenes*, which is present in this mutant on a carbohydrate-poor diet. This in turn leads to the longer survival of this mutant, comparable to control flies. A carbohydrate-rich diet can rescue the glycogen loss in flies with lowered ADGF-A but the pathogen load, being intracellular, is still increased and survival is as short as on a carbohydrate-poor diet. It seems that increased dietary glucose can compensate for the glycogen loss detected on a carbohydrate-poor diet, which is potentially associated with the nutrient exploitation by the pathogen in these flies, as discussed above. However, the higher pathogen burden still kills the flies faster, suggesting that the survival is primarily determined by the pathogen number.

Although we focus on phagocytosis in this work, there are other immunity mechanisms which may influence the host resistance and physiology. While melanization was not observed with *S*. *pneumoniae* infection, it was shown to play an important role in controlling the extracellular population of *L*. *monocytogenes* [22]. Therefore, the increased extracellular *L*. *monocytogenes* load detected in *adoR* could also be due to the lower induction of melanization response. However, we detected a rather stronger disseminated melanization [22] in the *adoR* mutant suggesting that the induction of melanization is not lowered by the lack of adenosine signaling and may rather reflect the reaction provoked to a greater extent by the increased extracellular population of *L*. *monocytogenes* in this mutant. Nevertheless, the role of adenosine signaling in the melanization arm of immunity requires further work.

Host immunity is also mediated by the production of antimicrobial peptides (AMPs). We did not detect clear and consistent differences in the expression of AMPs in the mutants that would be in agreement with the observed effects on resistance, i.e. lower expression of AMPs in *adoR* and higher expression in *adgf-a/+*. The expression of AMPs was shown to be dependent on pathogen load, at least with *L*. *monocytogenes* [19]. We did not test the expression with different loads and therefore we can directly compare the expressions between mutants and control at only 6 hpi when the pathogen loads are similar among the genotypes both for *S*. *pneumoniae* and *L*. *monocytogenes*. In the case of Defensin, Diptericin, and Drosocin, but not Metchnikowin, the *adoR* mutant showed lower expression during *S*. *pneumoniae* infection, which would be consistent with its lower resistance. However, such a difference was not detected at 18 hpi and in the case of *L*. *monocytogenes* for neither time point. In case of *adgf-a/+*, the expression of AMPs is mostly lower compared to control, including cases when the pathogen loads are comparable, which is in contrast to the observed higher resistance. While we cannot exclude the role of AMPs in the observed effects on resistance, this arm of immunity does not seem to play as important a role as phagocytosis.

Our work demonstrates the crucial role of e-Ado in mediating the systemic metabolic switch, which is required for effective immune response. Although we did not directly measure e-Ado levels, the opposite effects of the *adoR* mutation, which blocks adenosine signaling on one hand, and lowering ADGF-A, which degrades adenosine, on the other, leave little doubt that the observed effects are indeed associated with the e-Ado action. Being able to mount an adequate immune response is vital for the organism (as demonstrated by the lower resistance of *adoR*) but its regulation is as important as the response itself; reducing regulation by lowering ADGF-A may prolong the switch associated with a greater loss of the host energy reserves and may potentially lead to the exploitation of released nutrients by the pathogen. The regulatory role of ADGF-A is demonstrated by a hyperglycemic peak and consecutive decrease after 9–12 hpi during *S*. *pneumoniae* infection which coincides with a rise in ADGF-A expression; when one copy of ADGF-A gene is removed or ADGF-A is knocked down by RNAi, the hyperglycemia continues past this time point and glycogen continues to fall. It is important to note that ADGF-A was knocked down specifically only in hemocytes by the Hml-Gal4 driver; also, our hemocyte-specific expression analysis demonstrated that hemocytes are the primary source of ADGF-A. Therefore, the immune cells are the regulators of e-Ado actions during the immune response. This is in agreement with our previous results showing a specific expression of ADGF-A in specialized larval immune cells, lamellocytes that encapsulate parasitoid wasp eggs [29], which represent a later phase of immune response. Taken together with immune cells first releasing adenosine to usurp energy from the rest of the organism during a parasitoid wasp attack [11], and later producing ADGF-A to downregulate adenosine, we can say that the immune system is able to regulate its privileged access to nutrients by producing a regulator of the signal mediating the systemic metabolic switch.

**Fig 10:**
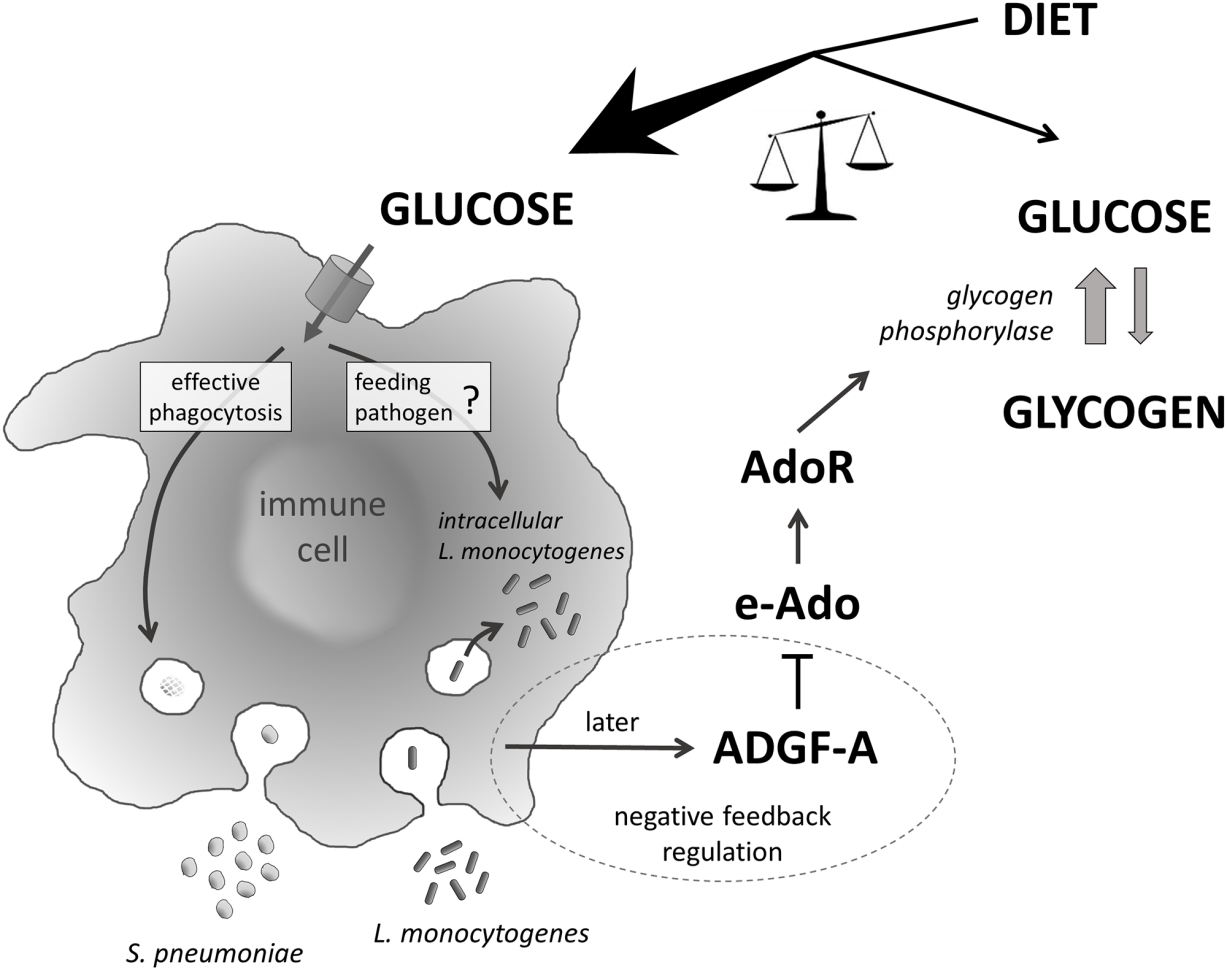
Model of energy regulation by extracellular adenosine during immune response and its impact on host-pathogen interaction. Extracellular adenosine (e-Ado) mediates a systemic metabolic switch via the adenosine receptor AdoR upon infection, leading to increased circulating glucose at the expense of glycogen storage. Glucose is required for an effective immune response but energy resources are limited and the energy may also be exploited by the pathogen. Therefore it is important to regulate e-Ado action, which is achieved by immune cells themselves expressing the e-Ado regulator ADGF-A. Lowering such regulation leads to an increased resistance to *S*. *pneumoniae* on one hand but also to lower glycogen stores and to an increased intracellular *L*. *monocytogenes* loads (possibly by feeding pathogen) on the other.

In summary (Fig 10), we demonstrate here that bacterial infections of the adult Drosophila flies are associated with a systemic metabolic switch, manifested by a hyperglycemia at the expense of glycogen stores. This switch supplies the immune system with the energy required for an effective response and is mediated by e-Ado signaling. Blocking e-Ado signaling demonstrates its crucial role for an effective immune response, in this case phagocytosis. However, the proper regulation of e-Ado by adenosine deaminase ADGF-A is also important. Although lowering such regulation may increase host resistance to some infections, it may also lead to an excessive loss of energy reserves during chronic infection. An increased release of energy allocated for the immune system may also be exploited by the pathogen leading to decreased host survival. Here we build on our previous work showing that the privileged behavior of the immune system is crucial for host resistance by revealing a mechanism in which the same immune system limits its own privilege to ultimately protect the whole organism.

## Materials and methods

### Fly stocks and culture

The fly strains used for manipulating adenosine were first backcrossed 10 times into the same *w*^*1118*^ strain with a genetic background based on CantonS, which was used as a control (referred to simply as *w* in text). To block adenosine signaling, we used a homozygous *adoR*^*1*^ null mutation (FBal0191589) of the adenosine receptor *AdoR* (CG9753; FBgn0039747). To enhance adenosine effects, we lowered the fly adenosine deaminase ADGF-A (CG5992; FBgn0036752) by heterozygous null *adgf-a*^*kar*^*/+* mutation [23], referred to as *adgf-a/+* in the text, and by RNA interference for the *ADGF-A* gene *P{GD17237}* (VDRC-50426; FBtp0028959) using the HmlΔ-Gal4 driver (FBti0128549) specific to hemocytes in combination with a thermosensitive GAL80 construct *P{tubP-GAL80*^*ts*^*}2* (FBti0027797). To induce RNAi, we crossed *w*^*1118*^; *HmlΔ-Gal4; P{tubP-GAL80*^*ts*^*}* flies to *w*^*1118*^; *ADGF-A*^*RNAi*^*-P{GD17237}* flies and the resulting progeny (referred to as *Hml>ADGF-A[RNAi]*) lowered *ADGF-A* mRNA to 20% or less of control levels (Fig 9A). As a control for RNAi, we crossed *w*^*1118*^; *ADGF-A*^*RNAi*^*-P{GD17237}* to *w*^*1118*^, with the resulting progeny referred to as *ADGF-A[RNAi]/+*. To induce RNAi for glycogen phosphorylase, we crossed y, *w*^*1118*^; *P{KK100434}VIE-260B* line (VDRC-109596; FBtp0066083) to either *P{ppl-GAL4*.*P}* (FBti0163688) or *Act-Gal4* in combination with a thermosensitive GAL80 construct *P{tubP-GAL80*^*ts*^*}* to obtain *P{KK100434}VIE-260B*/*P{ppl-GAL4*.*P}; P{tubP-GAL80*^*ts*^*}/+* (labeled as *ppl>GlyP[RNAi]*) or *P{KK100434}VIE-260B*/*P{tubP-GAL80*^*ts*^*}; Act-Gal4/+* (labeled as *Act>GlyP[RNAi]*) flies. KK control line *y*,*w*^*1118*^; *P{attP*,*y[+]*,*w[3`]}* (VDRC-60100) was crossed to the Gal4 lines to obtain control flies for the GlyP RNAi with the same genetic background (labeled as either *ppl x KK control* or *Act x KK control*). For hemocyte counting, we used the *HmlΔ-Gal4 UAS-EGFP* marker on chromosome II in each of the *w*, *adoR*, and *adgf-a/+* backgrounds. Flies were grown on cornmeal medium (8% cornmeal, 5% glucose, 4% yeast, 1% agar) in 6-oz square bottom plastic bottles (20 females per bottle laid eggs for 24 h only to prevent crowding). The *w*, *adoR* and *adgf-a/+* flies were raised in bottles at 25°C, 70% humidity with 12/12 hours light/dark cycle; the *Hml>ADGF-A[RNAi]*, *ppl>GlyP[RNAi]* and *Act>GlyP[RNAi]* and *ADGF[RNAi]/+* and *KK* control flies were raised at 18°C to suppress RNAi during development and transferred to 25°C 24 h before infection to induce RNAi. Two-day-old male progeny flies were anesthetized with carbon dioxide and collected in plastic vials (20 flies per vial) with either carbohydrate-poor, 0%-glucose (8% cornmeal, 4% yeast, 1% agar, no additional sugar) or carbohydrate-rich, 10%-glucose (8% cornmeal, 4% yeast, 10% glucose, 1% agar) diets and transferred every other day to a fresh meal.

### Bacterial strains and culturing conditions

The *Listeria monocytogenes* strain 10403S was stored at -80°C in brain and heart infusion (BHI) broth containing 25% glycerol. For the experiments bacteria were streaked onto Luria Bertani (LB) agar plates containing 100 μg/mL streptomycin and incubated at 37°C overnight; plates were stored at 4°C and used for inoculation for a period of two weeks. Single colonies were used to inoculate 3 mL of BHI and incubated overnight at 37°C without shaking to obtain a morning 600 nm optical density (OD600) of approx. 0.4. Further, *L*. *monocytogenes* cultures were diluted to OD600 0.01 in phosphate buffered saline (PBS) and stored on ice prior to loading into an injection needle. The *Streptococcus pneumoniae* strain EJ1 (D39 streptomycin-resistant derivative; [30]) was stored at -80°C in Tryptic Soy Broth (TSB) media containing 10% glycerol. For the experiments, bacteria were streaked onto blood-containing TSB agar plates containing 100 μg/mL streptomycin and incubated at 37°C overnight; a fresh plate was prepared for each experiment. Single colonies were used to inoculate 3 mL of TSB with 100 000 units of catalase (Sigma C40) and incubated overnight at 37°C + 5% CO2 without shaking. Morning cultures were 2x diluted in TSB with fresh catalase and were grown for an additional 4 hours, reaching an approximate 0.4 OD600. Final cultures were concentrated by centrifugation and re-suspended in phosphate buffered saline (PBS) so that the concentration corresponded to OD600 2.4 and stored on ice prior to injection needle loading. For sublethal doses, we used approximately 12000–15000 CFUs, for lethal doses 20 000 CFUs; the EC50 was between 15000–20000 CFU.

### Fly injection

Seven-day-old male flies were anaesthetized with carbon dioxide. The Eppendorf Femtojet microinjector and a drawn glass needle was used to inject precisely 50 nl of bacteria or mock buffer into the fly at the cuticle on the ventrolateral side of the abdomen. Infectious doses were determined for each experiment by plating a subset of flies at time zero. 50 nl of 10% 0.5-μm carboxylate-modified polystyrene (latex) beads (Sigma L5530) in PBS or pure PBS as control were injected 24 hour before infection into the adult fly body cavity to block phagocytosis.

### Pathogen load measurement

Single flies were homogenized in PBS using a motorized plastic pestle in 1.5 ml tubes. Bacteria were plated in spots onto LB (*L*. *monocytogenes*) or TSB (*S*. *pneumoniae*) agar plates containing streptomycin in serial dilutions and incubated overnight at 37°C before manual counting. To determine intracellular *L*. *monocytogenes* loads, flies were injected with 50 nl of gentamycin solution (1 mg/ml in PBS) 3 h prior to fly homogenization. Pathogen loads of 16 flies were determined for each genotype and treatment in each experiment; at least three independent infection experiments were conducted and the results were combined into one graph (in all presented cases, individual experiments showed comparable results). Values were transformed to logarithmic values, since they followed the lognormal distribution, and compared using unpaired t-tests corrected for multiple comparisons using the Holm-Sidak method in the Graphpad Prism software.

### Survival analysis

A total of 200 to 300 flies were injected for each genotype and treatment in one experiment; at least three independent infection experiments were repeated and combined into one survival curve (in all presented cases, individual experiments showed comparable results). Injected flies were placed into vials with 20 flies per vial, transferred to a fresh vial every other day and checked daily to determine mortality. Flies infected by *L*. *monocytogenes* were kept at 25°C and flies infected with *S*. *pneumoniae* were kept at 29°C. Survival curves were generated by Graphpad Prism software and analyzed by Log-rank and Gehan-Breslow-Wilcoxon (more weight to deaths at early time points) tests, as specified in the appropriate figure legends.

### Hemocyte counting and phagocytosis analysis

The number of hemocytes in adult flies were determined by counting Hml>GFP-positive cells visualized by confocal microscopy. The number of phagocytic cells was determined by injection of the marker pHrodo Red *S*. *aureus* Bioparticles (ThermoFisher Scientific) 40 min prior to fixing flies. Flies were fixed by 4% paraformaldehyde in PBS and imaged using confocal microscopy with maximal projection from five different layers; the same setting of the Z- stack range as well as the intensity of lasers were used for all animals. The cells were observed in whole flies to detect possible gross changes but no obvious differences were observed. The exact number of cells were counted within a selected thorax region as depicted in Fig 2A using Fiji software and compared by student t-tests using the Graphpad Prism software.

### Hemocyte isolation by fluorescent activated cell sorter

A fluorescent activated cell sorter was used for an isolation of *HmlΔ-Gal4 UAS-EGFP*-labeled hemocytes from the adult flies. Approximately 12 000 living cells were seperated from the homogenate of 100 fly males. The males used for this analysis were anesthetized with CO_2_, washed several times with PBS and then homogenized by sterile pestle in 800 μl of PBS. Cell homogenate was then filtered through a 70-μm cell strainer (Corning) and washed three times with ice cold PBS followed each time by centrifugation at 5000 RPM for 3 min at 4°C. Samples were filtered once more through a 40-μm cell strainer immediately before sorting. S3E cell sorter (BioRad) was used for sorting. GFP-specific cells constituted approximately 1% of the total cell number. Sorted hemocytes were verified by fluorescent microscopy and with DIC.

### Gene expression

Gene expression was analyzed by quantitative real-time PCR. Whole flies or sorted *HmlΔ-Gal4 UAS-EGFP* hemocytes were homogenized and total RNA was isolated by Trizol reagent (Ambion) according to manufacturer’s protocol. DNA contamination was removed by using a Turbo DNAse free kit (Ambion) according to the protocol (37°C 30 min) with subsequent inactivation of DNAse by DNAse inactivation reagent (5 min at RT, spin 13000 RPM at RT). Reverse transcription was done by Superscript III reverse transcriptase (Invitrogen) and amounts of mRNA of particular genes were quantified using the IQ Sybr Green Supermix Mastermix (BioRAd) on a CFX 1000 Touch Real time cycler (BioRad). Expressions were analyzed using double delta Ct analysis, normalized to the expression of Ribosomal protein 49 (Rp49) in the same sample. Relative values (fold change) to control (specified in each graph) were compared and shown in the graphs. Primer sequences can be found in S1 Table. Samples were collected from three independent infection experiments with three technical replicates for each experiment and compared by unpaired t-tests using Graphpad Prism software.

### Metabolite measurement

Glucose and glycogen were measured by approaches published in [31]. 3 flies were homogenized in 1x PBST (PBS with 0.3% Tween) and large tissue fragments were pelleted by centrifugation (800xg, 5 min, 4°C); half of the sample was used for protein quantification and the remainder was denatured by heating at 75°C for 10 min and stored at -80°C. Glucose was determined using a GAGO-20 kit (Sigma) according to the supplier’s protocol using spectrophotometric measurement at 540 nm. Glucose measurements probably also contained trehalose since this carbohydrate is usually present in flies, but we were not able to distinguish between glucose and trehalose in our measurements, most likely due to endogenous trehalase activity in homogenized samples. Glycogen samples were first treated with amyloglucosidase enzyme (Sigma) for 30 min. Protein concentration was analyzed by Bradford measurement. Samples were homogenized and proteins were dissolved in 1x PBS. A 100-μl volume of protein sample was mixed with 10 μl of Bradford solution (10 mg of Brilliant blue, 5 ml of 96% Ethanol, and 10 ml of 85% Phosphoric acid in 100 ml of solution). The concentration of proteins was derived from absorbance of the reaction solution at 595 nm. Values were compared by multiple unpaired t-tests using the Graphpad Prism software.

## Supporting information

S1 Fig Pathogen loads during 138 hours post-infection by *Streptococcus pneumoniae*. Dot-plot graphs of pathogen loads on a sugar-poor, 0%-glucose diet at 29°C. Values are shown by linear scale in thousands of CFU per fly (one dot represents one fly). Control w (black) are shown with the *adoR* mutant (red) and the *adgf-a/+* heterozygous mutant (green). Control *ADGF-A[RNAi]* (blue) are shown with *Hml>ADGF-A[RNAi]* (orange).
(TIF)

S2 Fig Pathogen loads during 24 hours post-infection by *Streptococcus pneumoniae*. Dot-plot graphs of pathogen loads on a sugar-poor, 0%-glucose diet at 29°C shown by logarithmic scale. Control *w* (black) are shown with the *adoR* mutant (red) and the *adgf-a/+* heterozygous mutant (green). Control *ADGF-A[RNAi]* (blue) are shown with *Hml>ADGF-A[RNAi]* (orange).
(TIF)

S3 Fig Pathogen loads in *adgf-a adoR* double mutant 24 hours post-infection by *Streptococcus pneumoniae*. Dot-plot graph of pathogen loads on a sugar-poor, 0%-glucose diet at 29°C at 0 and 24 hpi shown by logarithmic scale. Control *w* (black) are shown with the single *adoR* mutant (red) and the *adgf-a adoR/adoR* double mutant (olive).
(TIF)

S4 Fig Carbohydrate metabolism during *Listeria monocytogenes* infection. Free glucose levels (A) and glycogen levels (B) in PBS injected flies (dashed line) and in *L*. *monocytogenes* (L.m.) infected flies (solid line) of *w* control (black) and *adgf-a/+* (green) heterozygous mutant within 72 hpi. Lines connect mean values with SEM as error bars; no significant changes were detected (tested by unpaired t-test).
(TIF)

S5 Fig Pathogen load during 24 hours post-infection by *Listeria monocytogenes*. Dot-plot graphs of pathogen loads of *L*. *monocytogenes* in CFU per fly shown in logarithmic scale (values follow lognormal distribution) within the first 24 hpi; each dot represents one fly. (A) Total number of bacteria and (B) the number of intracellular bacteria after gentamicin treatment eradication of the extracellular population (marked as GENT). (C) Table showing P-values when comparing pathogen loads of various genotypes as indicated by the heading line at different time points (left-most column). These values were compared using unpaired t-tests corrected for multiple comparisons using the Holm-Sidak method. Red coloring represents significant values.
(TIF)

S6 Fig Disseminated melanization upon *Listeria monocytogenes* infection. Melanization was determined at day 7 post infection as black spots under the cuticle. Examples of small localized melanization in control *w* flies (left) and extensive melanization in the *adoR* mutant (right) were photographed on the dorsal side of the abdomen using a stereomicroscope equipped with a digital camera.
(TIF)

S7 Fig Survival of *Streptococcus pneumoniae* infection on carbohydrate-poor and carbohydrate-rich diets. Survival upon *S*. *pneumoniae* infection was investigated for flies on carbohydrate-poor, 0%-glucose (marked as 0%; solid lines) and carbohydrate-rich, 10%-glucose (marked as 10%; dashed lines) diets and were analyzed by both Log-rank and Gehan-Breslow-Wilcoxon tests. The *adoR* mutation (red) significantly reduces survival (P<0.0001) on a 0%-glucose diet compared to the w control. The survival of *adoR* is significantly improved by a 10%-glucose diet (P<0.0001 when compared to *adoR* on 0% and 10%-diets; marked by asterisks).
(TIF)

S8 Fig Gene expression analysis of antimicrobial peptides during infection. Expression of antimicrobial peptides Defensin, Diptericin, Drosocin and Metchnikowin in PBS-injected flies (PBS control, grey column) and those infected either by *S*. *pneumoniae* (S.p., black columns) on left panels or *L*. *monocytogenes* (L.m., black columns) on right panels, 6 and 18 hpi in four genotypes–*w* (as a control), *adoR* (blocking adenosine signaling), heterozygous *adgf-a/+* mutant and Hml-driven ADGF-A RNAi (*Hml>ADGF-A[RNAi]*), both for enhancing e-Ado effects. Bars show mean fold change relative to the expression of uninfected *w* control flies at 6 hpi obtained by qRT-PCR with SEM as error bars; stars mark significant changes in infected samples when compared to infected *w* control at the given time tested by two-way ANOVA.
(TIF)

S1 Table Primer sequences for gene expression analysis. (DOCX)

## References

[1] Palsson-McDermott EM, O’Neill LAJ. The Warburg effect then and now: From cancer to inflammatory diseases: Review essays. BioEssays. 2013;35: 965–973. doi: 10.1002/bies.201300084 24115022 10.1002/bies.201300084

[2] Spies CM, Straub RH, Buttgereit F. Energy metabolism and rheumatic diseases: from cell to organism. Arthritis Res Ther. 2012;14: 216–216. doi: 10.1186/ar3885 22747923 10.1186/ar3885 PMC3446535

[3] Gerriets VA, MacIver NJ. Role of T cells in malnutrition and obesity. Inflammation. 2014;5: 379 doi: 10.3389/fimmu.2014.00379 10.3389/fimmu.2014.00379 PMC4127479 25157251

[4] Passalacqua KD, Charbonneau M-E, O’Riordan MXD. Bacterial metabolism shapes the host:pathogen interface. Microbiol Spectr. 2016;4 doi: 10.1128/microbiolspec.VMBF-0027-2015 10.1128/microbiolspec.VMBF-0027-2015 PMC4922512 27337445

[5] Ayres JS, Schneider DS. The role of anorexia in resistance and tolerance to infections in Drosophila. PLoS Biol. 2009;7: e1000150–e1000150. doi: 10.1371/journal.pbio.1000150 19597539 10.1371/journal.pbio.1000150 PMC2701602

[6] Wang A, Huen SC, Luan HH, Yu S, Zhang C, Gallezot J-D, et al Opposing Effects of Fasting Metabolism on Tissue Tolerance in Bacterial and Viral Inflammation. Cell. 2016;166: 1512–1525.e12. doi: 10.1016/j.cell.2016.07.026 27610573 10.1016/j.cell.2016.07.026 PMC5555589

[7] Dionne MS, Pham LN, Shirasu-Hiza M, Schneider DS. Akt and FOXO dysregulation contribute to infection-induced wasting in Drosophila. Curr Biol. 2006;16: 1977–1985. doi: 10.1016/j.cub.2006.08.052 17055976 10.1016/j.cub.2006.08.052

[8] Straub RH. Insulin resistance, selfish brain, and selfish immune system: an evolutionarily positively selected program used in chronic inflammatory diseases. Arthritis Res Ther. 2014;16: S4 doi: 10.1186/ar4688 25608958 10.1186/ar4688 PMC4249495

[9] Straub RH, Schradin C. Chronic inflammatory systemic diseases—an evolutionary trade-off between acutely beneficial but chronically harmful programs. Evol Med Public Health. 2016; eow001 doi: 10.1093/emph/eow001 10.1093/emph/eow001 PMC4753361 26817483

[10] Eisenreich W, Heesemann J, Rudel T, Goebel W. Metabolic host responses to infection by intracellular bacterial pathogens. Front Cell Infect Microbiol. 2013;3 doi: 10.3389/fcimb.2013.00024 10.3389/fcimb.2013.00024 PMC3705551 23847769

[11] Bajgar A, Kucerova K, Jonatova L, Tomcala A, Schneedorferova I, Okrouhlik J, et al Extracellular Adenosine Mediates a Systemic Metabolic Switch during Immune Response. PLoS Biol. 2015;13: e1002135 doi: 10.1371/journal.pbio.1002135 25915062 10.1371/journal.pbio.1002135 PMC4411001

[12] Antonioli L, Csóka B, Fornai M, Colucci R, Kókai E, Blandizzi C, et al Adenosine and inflammation: what’s new on the horizon? Drug Discov Today. 2014;19: 1051–1068. doi: 10.1016/j.drudis.2014.02.010 24607729 10.1016/j.drudis.2014.02.010

[13] Davis JM, Zhao Z, Stock HS, Mehl KA, Buggy J, Hand GA. Central nervous system effects of caffeine and adenosine on fatigue. Am J Physiol—Regul Integr Comp Physiol. 2003;284: R399–R404. doi: 10.1152/ajpregu.00386.2002 12399249 10.1152/ajpregu.00386.2002

[14] Collis MG. The vasodilator role of adenosine. Pharmacol Ther. 1989;41: 143–162. doi: 10.1016/0163-7258(89)90104-6 2652149 10.1016/0163-7258(89)90104-6

[15] Shimkets LJ, Dworkin M. Excreted adenosine is a cell density signal for the initiation of fruiting body formation in Myxococcus xanthus. Dev Biol. 1981;84: 51–60. doi: 10.1016/0012-1606(81)90369-9 6788625 10.1016/0012-1606(81)90369-9

[16] Pham LN, Dionne MS, Shirasu-Hiza M, Schneider DS. A Specific Primed Immune Response in Drosophila Is Dependent on Phagocytes. PLOS Pathog. 2007;3: e26 doi: 10.1371/journal.ppat.0030026 17352533 10.1371/journal.ppat.0030026 PMC1817657

[17] Dolezal T, Dolezelova E, Zurovec M, Bryant PJ. A role for adenosine deaminase in Drosophila larval development. PLoS Biol. 2005;3: e201 doi: 10.1371/journal.pbio.0030201 15907156 10.1371/journal.pbio.0030201 PMC1135298

[18] Brand AH, Perrimon N. Targeted gene expression as a means of altering cell fates and generating dominant phenotypes. Dev Camb Engl. 1993;118: 401–415. 10.1242/dev.118.2.401 8223268

[19] Louie A, Song KH, Hotson A, Tate AT, Schneider DS. How Many Parameters Does It Take to Describe Disease Tolerance? PLOS Biol. 2016;14: e1002435 doi: 10.1371/journal.pbio.1002435 27088212 10.1371/journal.pbio.1002435 PMC4835111

[20] Chambers MC, Lightfield KL, Schneider DS. How the fly balances its ability to combat different pathogens. PLoS Pathog. 2012;8: e1002970–e1002970. doi: 10.1371/journal.ppat.1002970 23271964 10.1371/journal.ppat.1002970 PMC3521699

[21] Mansfield BE, Dionne MS, Schneider DS, Freitag NE. Exploration of host-pathogen interactions using Listeria monocytogenes and Drosophila melanogaster. Cell Microbiol. 2003;5: 901–911. doi: 10.1046/j.1462-5822.2003.00329.x 14641175 10.1046/j.1462-5822.2003.00329.x

[22] Ayres JS, Schneider DS. A Signaling Protease Required for Melanization in Drosophila Affects Resistance and Tolerance of Infections. PLOS Biol. 2008;6: e305 doi: 10.1371/journal.pbio.0060305 10.1371/journal.pbio.0060305 PMC2596860 19071960

[23] Dolezal T, Gazi M, Zurovec M, Bryant PJ. Genetic analysis of the ADGF multigene family by homologous recombination and gene conversion in Drosophila. Genetics. 2003;165: 653–66. 14573477 10.1093/genetics/165.2.653 PMC1462772

[24] Straub RH. Evolutionary medicine and chronic inflammatory state—known and new concepts in pathophysiology. J Mol Med. 2012;90: 523–534. doi: 10.1007/s00109-012-0861-8 22271169 10.1007/s00109-012-0861-8 PMC3354326

[25] Kelly B, O’Neill LA. Metabolic reprogramming in macrophages and dendritic cells in innate immunity. Cell Res. 2015;25: 771–784. doi: 10.1038/cr.2015.68 26045163 10.1038/cr.2015.68 PMC4493277

[26] Manzanillo PS, Ayres JS, Watson RO, Collins AC, Souza G, Rae CS, et al The ubiquitin ligase parkin mediates resistance to intracellular pathogens. Nature. 2013;501: 512–516. doi: 10.1038/nature12566 24005326 10.1038/nature12566 PMC3886920

[27] Chico-Calero I, Suárez M, González-Zorn B, Scortti M, Slaghuis J, Goebel W, et al Hpt, a bacterial homolog of the microsomal glucose-6-phosphate translocase, mediates rapid intracellular proliferation in Listeria. Proc Natl Acad Sci. 2002;99: 431–436. doi: 10.1073/pnas.012363899 11756655 10.1073/pnas.012363899 PMC117577

[28] Portnoy DA. A 20-year perspective on Listeria monocytogenes pathogenesis Listeria monocytogenes: Pathogenesis and Host Response. Springer; 2007 pp. 1–12. http://link.springer.com/chapter/10.1007/978-0-387-49376-3_1

[29] Novakova M, Dolezal T. Expression of Drosophila adenosine deaminase in immune cells during inflammatory response. PloS One. 2011;6: e17741 doi: 10.1371/journal.pone.0017741 21412432 10.1371/journal.pone.0017741 PMC3055890

[30] Joyce EA, Kawale A, Censini S, Kim CC, Covacci A, Falkow S. LuxS Is Required for Persistent Pneumococcal Carriage and Expression of Virulence and Biosynthesis Genes. Infect Immun. 2004;72: 2964–2975. doi: 10.1128/IAI.72.5.2964-2975.2004 15102809 10.1128/IAI.72.5.2964-2975.2004 PMC387900

[31] Tennessen JM, Barry W, Cox J, Thummel CS. Methods for studying metabolism in Drosophila. Methods. 2014;68: 105–115. doi: 10.1016/j.ymeth.2014.02.034 24631891 10.1016/j.ymeth.2014.02.034 PMC4048761

