# Supplementary figures and images for: Extracellular adenosine modulates host-pathogen interactions through regulation of systemic metabolism during immune response in Drosophila
Source: PLoS Pathog. 2018 Apr 27;14(4):e1007022. doi: 10.1371/journal.ppat.1007022 (PMC5942856; doi:10.1371/journal.ppat.1007022)

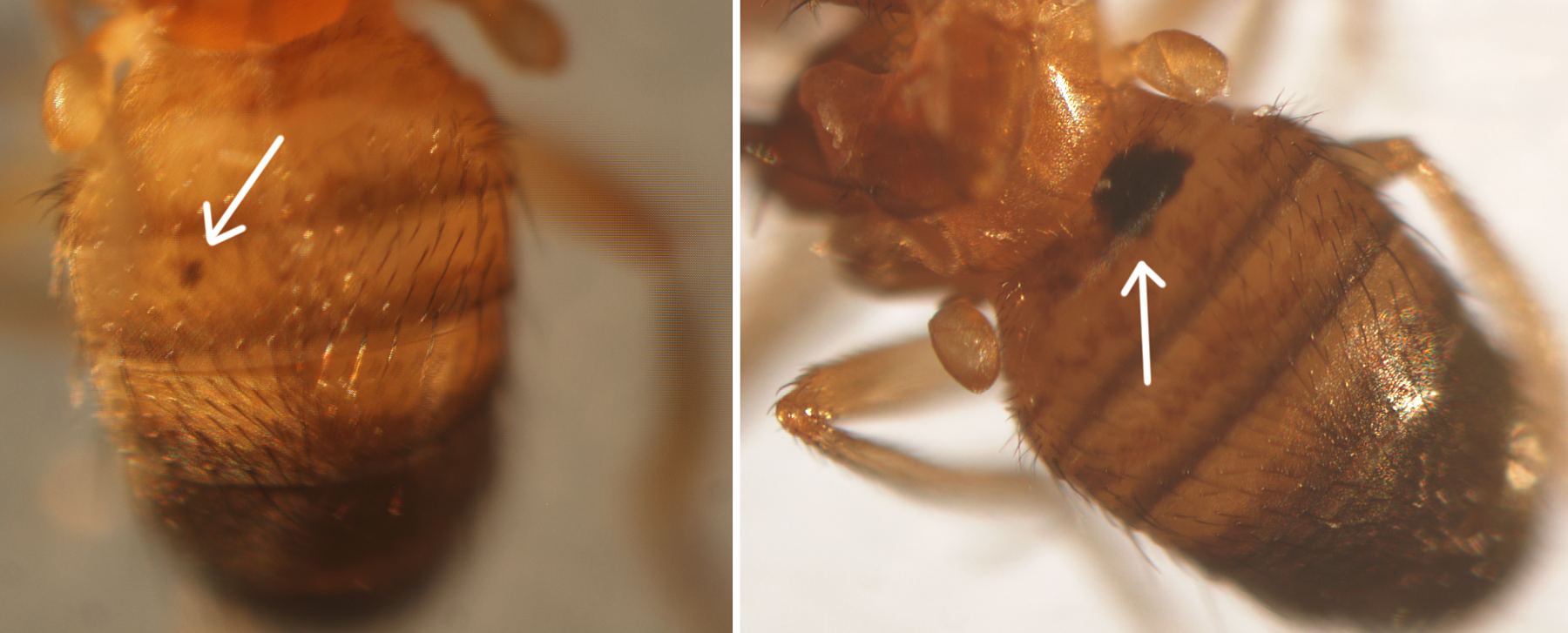

Supplement: S6 Fig — Melanization was determined at day 7 post infection as black spots under the cuticle. Examples of small localized melanization in control w flies (left) and extensive melanization in the adoR mutant (right) were photographed on the dorsal side of the abdomen using a stereomicroscope equipped with a digital camera. (TIF) [file ppat.1007022.s006.tif]
