# Supplementary material for: Extracellular adenosine modulates host-pathogen interactions through regulation of systemic metabolism during immune response in Drosophila
Source: PLoS Pathog. 2018 Apr 27;14(4):e1007022. doi: 10.1371/journal.ppat.1007022 (PMC5942856; doi:10.1371/journal.ppat.1007022)
Supplement: S1 Table — (DOCX) [file ppat.1007022.s009.docx]

| **Gene name** | **Gene number** | **Fly base ID number** | **Primer Fwd 5´-3´** | **Primer Rev 5´-3´** |
| --- | --- | --- | --- | --- |
| Glycogen phosphorylase | CG7254 | FBgn0004507 | CTTTATCTTCGGCATGACCG | GGCTTGGTTCTGGTAGGTC |
| Glycogen synthase | CG6904 | FBgn0266064 | CGATGAAGAAGTACAGGGTC | TATCATCGTTATTGCCTGGAG |
| Ribosomal protein 49 | CG7939 | FBgn0002626 | AAGCTGTCGCACAAATGGCG | GCACGTTGTGCACCAGGAAC |
| Ribosomal protein 49 | CG7939 | FBgn0002626 | GACGCTTCAAGGGACAGTATCTG | AAACGCGGTTCTGCATGAG |
| ADGF-A | CG5992 | FBgn0036752 | ATGTCATATAGCGTGGGAAC | ATGTGCGAGCCAAATACGG |
| Defensin | CG1385 | FBgn0010385 | GTTCTTCGTTCTCGTGG | CTTTGAACCCCTTGGC |
| Drosocin | CG10816 | FBgn0010388 | CCATCGTTTTCCTGCT | CCATCGTTTTCCTGCT |
| Diptericin A | CG12763 | FBgn0004240 | GCTGCGCAATCGCTTCTACT | TGGTGGAGTGGGCTTCATG |
| Metchnikowin | CG8175 | FBgn0014865 | AACTTAATCTTGGAGCGA | CGGTCTTGGTTGGTTAG |
